# Long-Term Enhancement of NMDA Receptor Function in Inhibitory Neurons Preferentially Modulates Potassium Channels and Cell Adhesion Molecules

**DOI:** 10.3389/fphar.2021.796179

**Published:** 2022-01-04

**Authors:** Dan Xia, Xinyang Zhang, Di Deng, Xiaoyan Ma, Samer Masri, Jianzheng Wang, Shaowen Bao, Songnian Hu, Qiang Zhou

**Affiliations:** ^1^ State Key Laboratory of Chemical Oncogenomics, Guangdong Provincial Key Laboratory of Chemical Genomics, Peking University Shenzhen Graduate School, Shenzhen, China; ^2^ International Institute for Translational Chinese Medicine, School of Pharmaceutical Sciences, Guangzhou University of Chinese Medicine, Guangzhou, China; ^3^ Department of Physiology, University of Arizona, Tucson, AZ, United States; ^4^ CAS Key Laboratory of Genome Sciences and Information, Beijing Institute of Genomics, Chinese Academy of Sciences, Beijing, China

**Keywords:** NMDAR-positive allosteric modulator, RNA-seq, electrophysiological recording, potassium channels, cell adhesion molecules

## Abstract

Effectively enhancing the activity of inhibitory neurons has great therapeutic potentials since their reduced function/activity has significant contributions to pathology in various brain diseases. We showed previously that NMDAR positive allosteric modulator GNE-8324 and M-8324 selectively increase NMDAR activity on the inhibitory neurons and elevates their activity *in vitro* and *in vivo.* Here we examined the impact of long-term administering M-8324 on the functions and transcriptional profiling of parvalbumin-containing neurons in two representative brain regions, primary auditory cortex (Au1) and prelimbic prefrontal cortex (PrL-PFC). We found small changes in key electrophysiological parameters and RNA levels of neurotransmitter receptors, Na^+^ and Ca^2+^ channels. In contrast, large differences in cell adhesion molecules and K^+^ channels were found between Au1 and PrL-PFC in drug-naïve mice, and differences in cell adhesion molecules became much smaller after M-8324 treatment. There was also minor impact of M-8324 on cell cycle and apoptosis, suggesting a fine safety profile.

## Introduction

Proper balance between excitation and inhibition (E/I) is critical to many vital functions of the brain and its alteration is believed to contribute to the pathogenesis of various brain diseases (Isaacson and Scanziani, 2011; Yizhar et al., 2011; Nelson and Valakh, 2015). Proper functioning of the inhibitory neurons plays a key role in tuning this E/I balance. A decreased function/activity in inhibitory neurons was found in various brain diseases, including schizophrenia, autism, Alzheimer’s diseases, and during aging (Nakazawa et al., 2012; Lewis, 2014; Palop and Mucke, 2016; Chen et al., 2020). Thus, effective enhancement of inhibitory neuron activity to rebalance E/I has great therapeutic values and is hotly pursued.

One most extensively studied inhibitory neuron type is the parvalbumin (PV)-containing neurons, which stand out from the other subtypes of inhibitory neurons due to their ability to spike at high frequencies and their critical contributions to working memory and gamma oscillation (Bartos et al., 2007; Sohal et al., 2009; Buzsaki and Wang, 2012). These PV-neurons provide powerful perisomatic inhibition to excitatory neurons to enable synchronized brain rhythmic activity between excitatory and inhibitory neurons within the neural network (Freund and Katona, 2007). Their dysfunction is also well documented in the literature, from reduced PV expression (Filice et al., 2016; Xia et al., 2021), altered synaptic excitability (Xiao et al., 2020), to cell loss (Sakai et al., 2008; Du and Grace, 2016). These alterations are intimately associated with drastic changes in the behavior of neural networks. The fast-spiking property of PV-neurons is enabled by coordinated synaptic properties and ion channels tailored to allow rapid recovery after spiking, which include fast kinetics of AMPA receptor, Na^+^ channels, and K^+^ channels (Hu et al., 2018). It is also well known that PV-neurons are vulnerable to oxidative stress which causes compromised cell health or even neuronal loss, as implicated in the pathophysiology of schizophrenia, bipolar disorder, and posttraumatic stress disorder (Lewis et al., 2005; Hardingham and Do, 2016; Steullet et al., 2017). Thus, how to maintain the proper functions of PV-neurons without compromising their health is a key issue to be addressed when improving their functions.

One important receptor to both physiological functions and pathological alterations in PV-neurons is the NMDA-subtype glutamatergic receptors (NMDAR) (Paoletti et al., 2013; Akgul and McBain, 2016). For example, NMDAR hypofunction has been proposed as a major drive for the pathogenesis of schizophrenia (Gonzalez-Burgos and Lewis, 2012; Nakazawa et al., 2017). One strategy to enhance inhibitory neuron activity is to correct or compensate NMDAR functions. Recent studies have shown that this can be effectively achieved using positive allosteric modulators (PAMs) of NMDARs (Hackos et al., 2016; Hackos and Hanson, 2017; Yao and Zhou, 2017; Yao et al., 2018). The reason for using a PAM rather than agonist of NMDARs is to avoid potential excitotoxicity (Hardingham and Bading, 2010; Ge et al., 2020). Our previous studies revealed that NMDAR-PAM GNE-8324 and its derivative M-8324 selectively enhance NMDARs on the inhibitory neurons both *in vitro* and *in vivo* (Hardingham and Bading, 2010; Yao et al., 2018; Deng et al., 2020; Ge et al., 2020). Acute administration of M-8324 results in reduced E/I ratio and enhanced sensory functions (Deng et al., 2020). However, whether long-term use of NMDAR-PAMs could induce potential side-effects such as synapse loss or even neuronal death is unknown. Deng (Deng et al., 2020) mostly focused on the impact of NMDAR-PAMs on the sensory processing due to its sensitivity to E/I balance in a dynamic manner, but equally important is the contribution of NMDARs on the inhibitory neurons in the prefrontal cortex (PFC) based on its prominent contributions to brain diseases. Since GNE-8324 and M-8324 enhance the activity of inhibitory neurons from the presynaptic side rather than from the postsynaptic site as is achieved with GABA receptor PAM benzodiazepine, not much is known about either functional or pathological consequence associated with this mode of action, especially after prolonged drug treatment. In addition, long-lasting alterations in the activity of inhibition of neurons have been shown to be associated with synaptic and homeostatic modifications (Tsodyks et al., 1997; Mitra et al., 2011; Turrigiano, 2011; Rogers et al., 2021).

There are three main aims of the current study: (1) to understand the long-term impact of inhibitory neuron-selective NMDAR-PAM M-8324 on the basic electrophysiological properties and functions of GABAergic and glutamatergic neurons; (2) to examine the long-term impact of M-8324 at the gene level; and (3) to compare the basal difference of PV-neurons between distinct brain regions. By using a combination of electrophysiological recordings (both *in vivo* and *in vitro*) and RNA-seq on PV-neurons, we found that key electrophysiological parameters and expression of glutamate receptors (GluRs), GABARs, Na^+^ channels, and Ca^2+^ channels were minimally altered by long-term M-8324 treatment or between different brain regions. However, large differences in cell adhesion molecules (CAMs) and K^+^ channels’ expression were seen in the above conditions. Our study provides important information for understanding PV-neurons’ physiological functions and pathological alterations, as well as the impact of long-term enhancement of NMDARs on the inhibitory neurons.

## Materials and Methods

### Animals

Mice were housed and fed at Peking University Shenzhen Graduate School. *PV:Cre* mice (B6; 129P2-Pvalb^tm1(cre)Arbr^/J, #008069) selectively express Cre recombinase in PV-neurons were bred with *Ai9* mice (Gt (ROSA)26Sor^tm9(CAG-tdTomato)Hze^, #007909) allowing for the Cre-dependent expression of red-fluorescent protein (tdTomato) under the *CAG* promoter selectively in PV-neurons. All experiments have been approved by the Peking University Shenzhen Graduate School Animal Care and Use Committee and were performed in accordance with the ARRIVE guidelines on the Care and Use of Experimental Animals.

### Osmotic Pump Implantation, Electrophysiological Recording, and Sensory Stimuli

Mice were anesthetized with isoflurane (∼1% in a gas mixture) in a stereotaxic apparatus. Osmotic pump (200 µL, Alzet, Model 2001) was filled with Vehicle (Veh), 100 µM, or 300 µM M-8324. The concentration of M-8324 used for RNA-seq was 300 µM. The infusion kit (Alzet, Brain Infusion Kit 3) and osmotic pump implantation were performed as described before (Deng et al., 2020). Infusion into the lateral ventricle was set to a rate of 1.0 μL/h for 1 week. Spontaneous and sound evoked spike rates were recorded in Veh, 100, and 300 µM M-8324 groups. The drug washout group was tested 7 days after the end of infusion. Electrophysiological recording and sensory stimulation were carried out as described before (Deng et al., 2020). Mice were euthanized with carbon dioxide after the completion of experiments. Spikes from individually recorded neurons were sorted using Plexon Offline Sorter. Sorted spikes were analyzed using Neuro Explorer. Excitatory and inhibitory neurons classification, E/I ratio calculation were conducted as previously described (Deng et al., 2020).

### Cell Harvesting

PV-neurons were identified using tdTomato fluorescence in brain slices. Weak negative pressure was applied to aspirate the entire neuron into the glass patch which was treated with DEPC water (ThermoFisher) in advance. The pipette tip was broken onto the wall of a 0.2 ml tight-lock tube (TubeOne) to allow the entire neuron to be immersed in a 1 μL drop of RNase-free lysis buffer [provided by Beijing Genomics institution (BGI)] placed on the side of the tube. The tube was kept in ice and two PV-neurons were collected into one tube within 5 min. The tube was then placed in dry ice until 20 PV-neurons were harvested from either primary auditory cortex (Au1) or prelimbic prefrontal cortex (PrL-PFC) of each mouse. Three mice were used for each brain region from either Veh or M-8324 group. Samples were rapidly spun down (5–10 s) and stored at −80°C before reverse transcription. Reverse transcription, PCR amplification, and sequencing were performed by BGI.

### RNA Sequencing

A total of 12 samples were sequenced. Only samples qualified for amplification by Agilent 2,100 Bioanalyzer (Agilent Technologies) entered the subsequent sequencing. Main peak of the amplified product is 1,000–2,000 bp, and samples with a concentration greater than 0.2 ng/μL were considered of adequate quality. After removing reads with low quality, linker contamination and excessively high N content of unknown bases, clean reads were aligned to the reference genome (*Mus_musculus*: NCBI_GCF_000001635.26_GRCm38. p6) (https://www.ncbi.nlm.nih.gov/assembly/GCF_000001635.26) using HISAT. Clean reads were aligned to the reference genes by using Bowtie2. Genes were also annotated in other functional databases: GeneBank, GO, KEGG, Interpro, and Pfam.

Differential expression analysis was conducted using DESeq2 package in R software (1.2.22). Differentially expressed genes (DEGs) were defined as those with |fold change| ≥2 and Q value (a corrected *p*-value using Benjamini and Hochberg multiple testing correction) <0.05. GO enrichment was conducted using Dr. Tom online system provided by BGI. Generally, GO functional terms of Q-value <0.05 is regarded as significant enrichment.

Notably, considering that genes with extremely low expression levels may have errors in calculating the significance of differences, and they may not significantly impact cellular functions, genes with expression levels (i.e., FPKM) less than 0.1 in all four groups (i.e., Au1-Veh, Au1-M-8324, PrL-Veh, and PrL-M-8324) were excluded from analysis.

### Slice Electrophysiology

Mice were deeply anesthetized with phenobarbital sodium and decapitated under anesthesia. Mouse brains were rapidly removed and placed in an ice-cold cutting solution containing (in mM): 110 choline chloride, 7 MgSO_4_, 2.5 KCl, 1.25 NaH_2_PO_4_, 25 NaHCO_3_, 25 D-glucose, 11.6 sodium ascorbate, 3.1 sodium pyruvate, and 0.5 CaCl_2_ gassed with 95% O_2_ and 5% CO_2_. Slices of 350 μm were cut with a DTK-1000 tissue slicer (DTK, Japan) in cutting solution. Slices were transferred to a holding chamber with artificial cerebrospinal fluid ACSF containing (in mM): 127 NaCl, 2.5 KCl, 1.25 NaH_2_PO_4_, 25 NaHCO_3_, 25 D-glucose, 2 CaCl_2_, and 1 MgSO_4_, and allowed to recover for 30 min at 32°C, then kept at room temperature for at least 1 h before recording.

Recordings were conducted in the PrL-PFC or Au1 slices on an Olympus microscope (BX51WI) with a×40 water-immersion differential interference contrast objective, at room temperature (23–26°C) with oxygenated ACSF (4–5 ml/min). Resistance of the recording pipette was 4–8 MΩ. Recording pipettes were filled with K^+^ gluconate based intracellular solution or Cs^+^-containing internal solution. K^+^-gluconate solution contains (in mM): 128 K^+^-gluconate, 10 NaCl, 2 MgCl_2_, 10 Hepes, 0.5 EGTA, 4 Na_2_ATP, and 0.4 NaGTP. Cs^+^-solution contains (in mM): 125 CsMeSO_4_, 5 NaCl, 1.1 EGTA, 10 HEPES, 0.3 Na_2_GTP, 4 Mg-ATP, and 5 QX-314. Recordings were made from layer II/III of the PrL-PFC or Au1, in a depth of 50–100 μm from slice surface. PV-neurons were identified by tdTomato fluorescence in *PV:Cre/Ai9* mice. HEKA EPC10 double patch clamp amplifier was used. Signals were collected at a sampling rate of 10 KHz and filtered at 2 KHz. Neurons with holding current >−200 pA (at −70 mV) were excluded from the data analysis. To record spontaneous excitatory post-synaptic currents (sEPSCs), somatic whole-cell voltage clamp recording (at −70 mV) was obtained from layer II/III PV-neurons in the PrL-PFC or Au1. To record spontaneous inhibitory post-synaptic currents (sIPSCs), whole-cell voltage clamp recording was made at +5 mV. All neurons were recorded for at least 5 min to collect sEPSCs or sIPSCs. Whole-cell current clamp recording of evoked spikes was done using a series of 500 ms depolarizing current pulses with 4 s intervals, and every step with an increase of 20 pA (from 0 to 380 pA).

We used a few parameters to examine AP properties. Rheobase current, defined as the amount of current required to elicit a single AP, was measured using Clamp fit. AP amplitude was calculated by measuring the absolute maximum amplitude from AP peak to −70 mV (holding Vm). Half-width was measured as the time difference between the rising phase and falling phase of the AP with amplitude one-half of the maximum AP amplitude. Afterhyperpolarization (AHP) was measured using AHP peak and latency. AHP peak is the difference in membrane potentials between AP threshold and trough (lowest point of an AP), while AHP latency is the time difference between AP peak and trough. The delay to the onset of first AP is calculated from the start of a depolarizing current step to the peak of first AP evoked by rheobase. More than 400 sEPSC or sIPSC events in each neuron were identified and analyzed using Mini Analysis software. Mean amplitude of sEPSC or sIPSC in each neuron was used for group analysis.

### Statistical Analysis and Graphing

Statistical significance was calculated using two-tailed paired/unpaired *t*-test, one-way/two-way RM ANOVA, as noted. Data are reported as mean ± SEM. Significance levels are noted as * (*p* < 0.05), ** (*p* < 0.01), *** (*p* < 0.001). Figures were generated using Dr. Tom online system, STRING (version 11.0), Cytoscape (version 3.8.2), Adobe Illustrator CC 2018, and Graphpad 9.0.

## Results

To understand the impact of long-term enhancing NMDARs and activity of GABAergic neurons *in vivo*, we recorded from genetically identified PV-neurons in brain slices from mice being treated with M-8324 for 7 days, recorded neuronal activity *in vivo*, and obtained RNAs from PV-neurons for RNA-seq analysis. We performed these experiments in two representative brain regions: Au1 which mainly processes auditory information and PFC which plays key roles in executive and higher integrative functions. Au1 also allows us to explore the impact of NMDAR-PAMs on sensory stimuli-evoked responses in addition to spontaneous neuronal activity. In addition to understand the impact of NMDAR-PAMs, we also examined the similarity and differences of PV-neurons in two distinct brain regions.

### *In vivo* Activity of Inhibitory and Excitatory Neurons After 7-days M-8324 Treatment

Our previous study showed that acute infusion of M-8324 into the brain ventricle enhances spiking of fast-spiking (FS, inhibitory neurons) neurons and reduces spiking of regular spiking (RS, mostly excitatory neurons) neurons in Au1, for both sound-evoked and spontaneous activity (Deng et al., 2020). To understand whether this activity profile persists after 7-days treatment with M-8324, we recorded both spontaneous and sound-evoked neuronal spiking using multi-electrode arrays in Au1 under Ketamine-Xylazine anesthetics, after 7-days infusion or 7-days infusion followed by 7-days drug withdrawal. In some experiments, we tested two concentrations of M-8234, low (100 μM) and high (300 μM).

**FIGURE 1:**
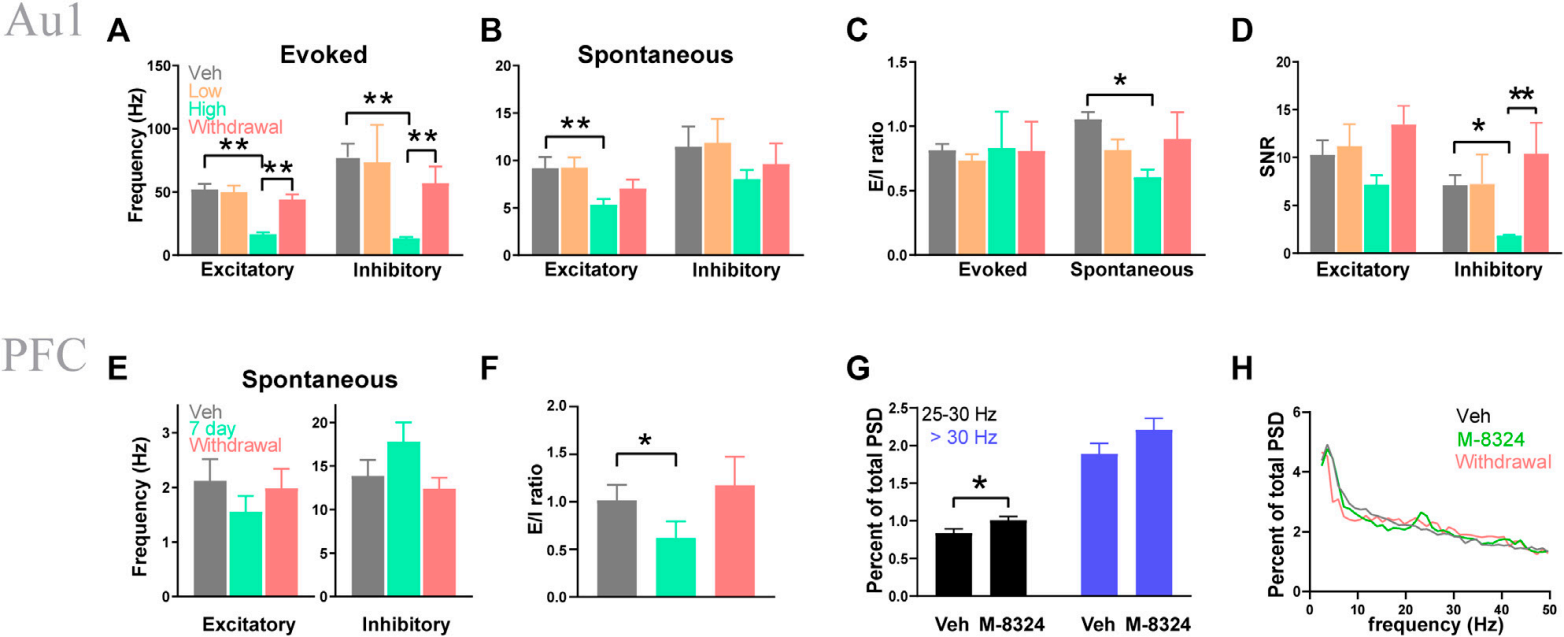
Seven-day infusion of M-8324 altered sound-evoked and spontaneous spiking in Au1 and PFC *in vivo*. **(A)** A significantly lower sound-evoked spike frequency in the high-dose group which recovered 1 week after M-8324 withdrawal in both excitatory and inhibitory neurons in Au1. **(B)** A significantly lower spontaneous spike frequency in excitatory neurons in the high-dose group in Au1. **(C)** A significantly higher E/I ratio in spontaneous spiking in excitatory neurons in the high-dose group. **(D)** A significantly lower signal-to-noise (SNR) in inhibitory neurons in the high-dose group. In **A**–**D**, for excitatory neurons, N = 70 cells/6 mice (vehicle), 49 cells/6 mice (M-8324 100 µM), 100 cells/6 mice (M-8324 300 µM), 73 cells/6 mice (withdrawal). For inhibitory neurons, N = 17 cells/6 mice (vehicle), 7 cells/6 mice (M-8324 100 µM), 25 cells/6 mice (M-8324 300 µM), 22 cells/6 mice (withdrawal). **(E)** Spontaneous spiking frequency did not change in inhibitory neuron in PFC after M-8324 7-days infusion. **(F)** E/I ratio was lower in PFC after M-8324 7-days infusion and recovered after withdrawal for 7 days. In **E**–**F**, for excitatory neurons, N = 24 cells/6 mice (vehicle), 24 cells/6 mice (7 days), 21 cells/6 mice (withdrawal). For inhibitory neurons, N = 10 cells/6 mice (vehicle), 10 cells/6 mice (7 days), 7 cells/6 mice (withdrawal). **(G)** Percent of high beta power spectral densities increased in M-8324 7-days infusion group. N = 6 mice for both groups. **(H)** Sample power spectral densities for vehicle, M-8324, and withdrawal groups.

Excitatory neurons (RS) and inhibitory neurons (FS) were distinguished using established criteria (Methods). A significantly lower frequency of sound-evoked spiking was seen in the high dose M-8324 group in both excitatory and inhibitory neurons, which recovered to pre-drug level after M-8324 withdrawal (Figure 1A). A lower spontaneous spiking was only seen in the excitatory neurons (Figure 1B). We computed E/I ratio by calculating the ratio of spike frequency of excitatory neurons over that of inhibitory neurons and found a significantly lower value only for the spontaneous activity due to a more prominent reduction in E (Figure 1C). For signal-to-noise ratio (SNR), a lower value was only seen in the inhibitory neurons in the high dose M-8324 group (Figure 1D). Taken together, these results showed: (1) differential impacts of M-8324 on the E/I ratio for spontaneous and evoked responses, (2) maintained E/I balance for evoked responses albeit alterations in E and I during the prolonged presence of M-8324, and (3) E/I ratio recovers after M-8324 withdrawal suggesting the absence of persistent effect. After testing low and high doses in Au1, in the subsequent PFC studies we have only used high dose M-8324.

We next recorded spontaneous activity from neurons in the layer 2/3 of PrL-PFC and separated excitatory from inhibitory neurons. We found no significant difference in spike frequency in the inhibitory neurons in the high dose M-8324 group (Figure 1E). There was also a significantly smaller E/I ratio during M-8324 infusion which recovered after drug washout (Figure 1F). Interestingly, the power of gamma oscillations was significantly higher in the M-8324 group for 25–30 Hz frequency range (Figures 1G,H), consistent with altered E/I ratio. Put together, these results show that changes induced by 7-days M-8324 infusion are partially similar to changes after acute infusion (Deng et al., 2020), and most of these changes are readily reversed after drug withdrawal.

### Distinct Electrophysiological Properties and Transcriptomes of PV-Neurons Between Au1 and PrL-PFC in Naive Mice

**FIGURE 2:**
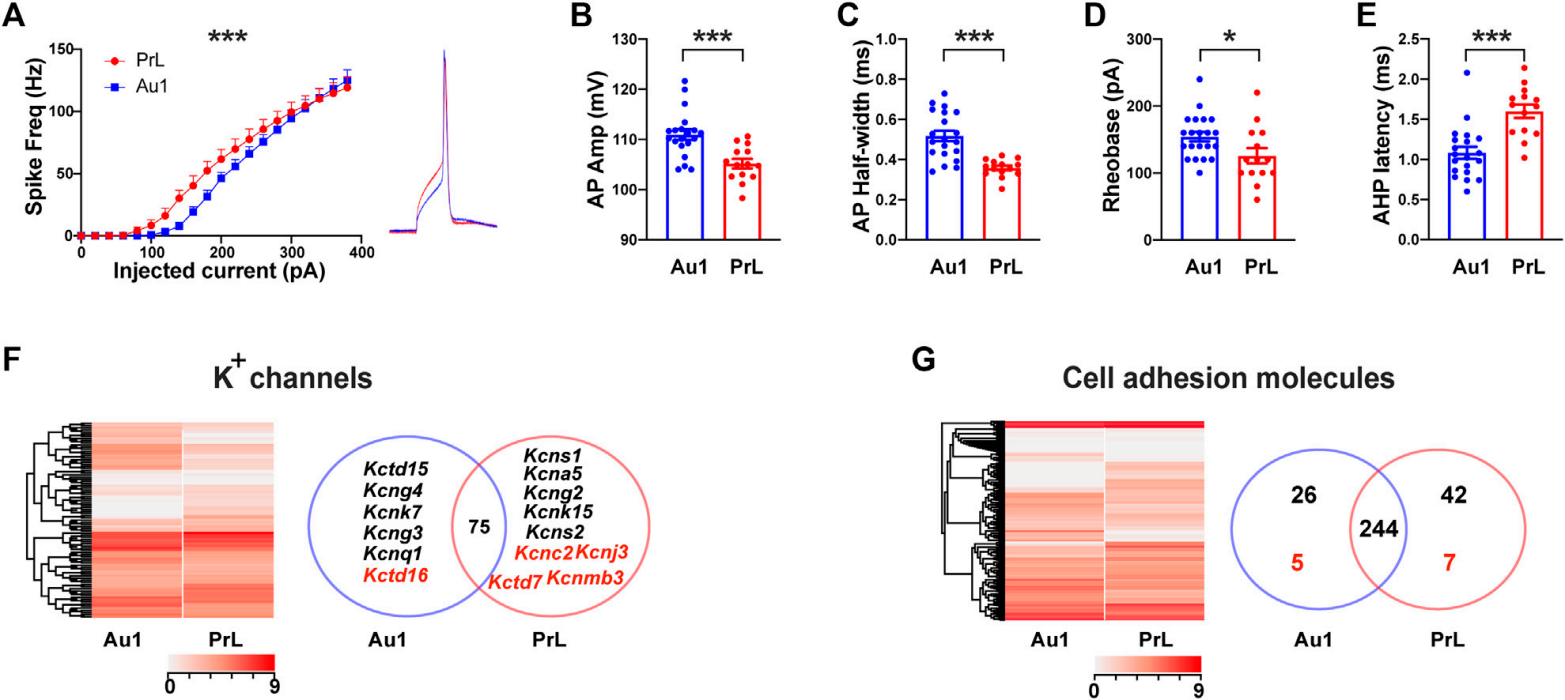
Electrophysiological properties and gene expression profiles in PV-neurons in Au1 and PrL-PFC. **(A)** A significantly higher intrinsic excitability in PV-neurons in the PrL-PFC than in those in Au1. **(Right)** Representative single spike in Au1 and PrL-PFC. **(B)** A significantly lower amplitude of action potentials in PrL-PFC PV-neurons compared to Au1. **(C)** A significantly lower half-width of action potentials in PrL-PFC PV-neurons compared to Au1. **(D)** A significantly lower rheobase of action potentials in PrL-PFC PV-neurons compared to Au1. **(E)** A significantly higher AHP latency of action potentials in PrL-PFC PV-neurons compared to Au1. **(F)** Comparisons of K^+^ channels gene expression between Au1 and PrL-PFC. **(Left)** Heatmap showing expression of all detected K^+^ channel subunits in Au1 and PrL-PFC. **(Right)** A Venn diagram showing Au1-specific or PrL-PFC-specific genes (names in black), and genes showed significantly higher expression in either brain region (names in red). Seventy-five genes not differentially expressed in Au1 and PrL-PFC. **(G)** Expression of cell adhesion molecules in Au1 and PrL-PFC. **(Left)** Heatmap showing expression of all detected CAMs. **(Right)** A Venn diagram showing the numbers of Au1-specific or PrL-specific CAMs (numbers in black), and genes showed significantly higher expression in either brain region (numbers in red). There are 244 genes expressed in both brain regions with no difference in expression level. For electrophysiology, N = 19 cells/3 mice for Au1 group, N = 14 cells/3 mice for PrL-PFC group. In all heatmaps, the redder the color the higher the expression level. Standardized method: log(value + 1). *p < 0.05. ***p < 0.001.

**FIGURE 3:**
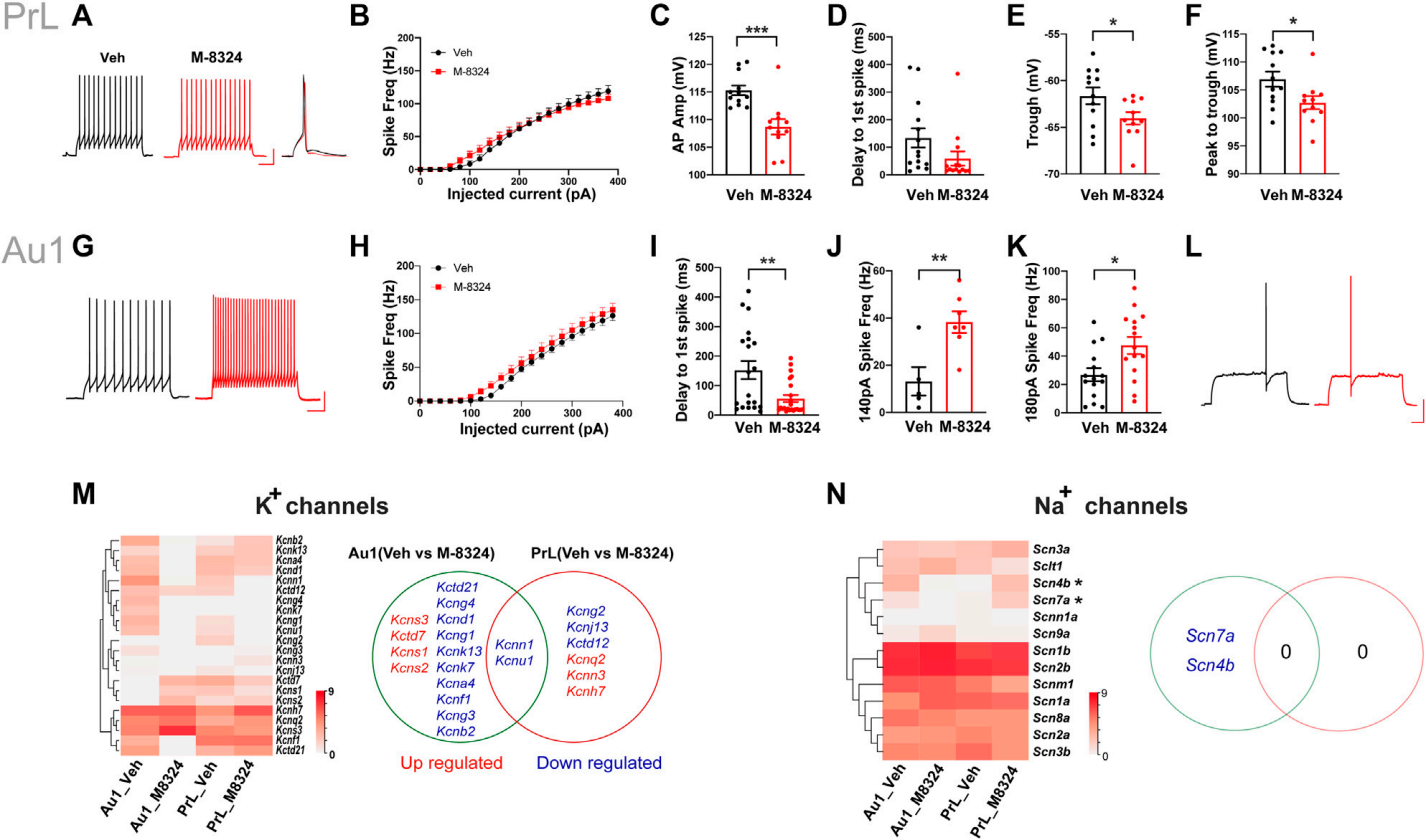
Changes in intrinsic excitability, spike properties, expression of potassium, and sodium channel genes in PV-neurons in Au1 and PrL-PFC after 7-days M-8324 treatment. **(A)** Representative spike patterns and single spike in PrL-PFC PV-neurons in Veh and M-8324 group. Current injection used, 160 pA and 500 msec. Scale bars, 25 mV/100 ms. **(B)** Intrinsic excitability of PV-neurons was not different between Veh and M-8324 groups in PrL-PFC. **(C)** A significantly lower amplitude of action potentials in PrL-PFC PV-neurons in M-8324 group compared to Veh group. **(D)** Delay to first spike was not different between M-8324 and Veh group in PrL-PFC. **(E)** A significantly lower trough of action potentials in PrL-PFC PV-neurons in M-8324 group compared to Veh group. **(F)** A significantly lower peak to trough of action potentials in PrL-PFC PV-neurons in M-8324 group compared to Veh group. **(G)** Representative spike patterns in Au1 PV-neurons in Veh and M-8324 group. Current injection used, 140 pA and 500 msec. Scale bars, 25 mV/100 ms. **(H)** Intrinsic excitability of PV-neurons was not different between Veh and M-8324 groups in Au1. **(I)** A significantly lower delay to first spike in Au1 PV-neurons in M-8324 group compared to Veh group. **(J)** A significantly higher spike frequency in Au1 PV-neurons in M-8324 group compared to Veh group with 140 pA current injection. **(K)** A significantly higher spike frequency in Au1 PV-neurons in M-8324 group compared to Veh group with 180 pA current injection. **(L)** Representative spike traces showing delay to first spike pattern from Au1 PV-neurons in Veh and M-8324 group. Current injection used, 120 pA and 500 msec. Scale bars, 25 mV/100 ms. **(M)** **(Left)** Heatmap of the differentially expressed genes of K^+^ channels between Veh and M-8324 groups in both Au1and PrL-PFC. **(Right)** A Venn diagram showing similarities and differences of the differentially expressed K^+^ channel genes after M-8324 treatment in Au1 and PrL-PFC. **(N)** **(Left)** Heatmap of all detected Na^+^ channel subunits in Veh and M-8324 groups in both Au1and PrL-PFC. **(Right)** A Venn diagram showing similarities and differences of differential Na^+^ channel subunits after M-8324 treatment in Au1 and PrL-PFC.

**FIGURE 4:**
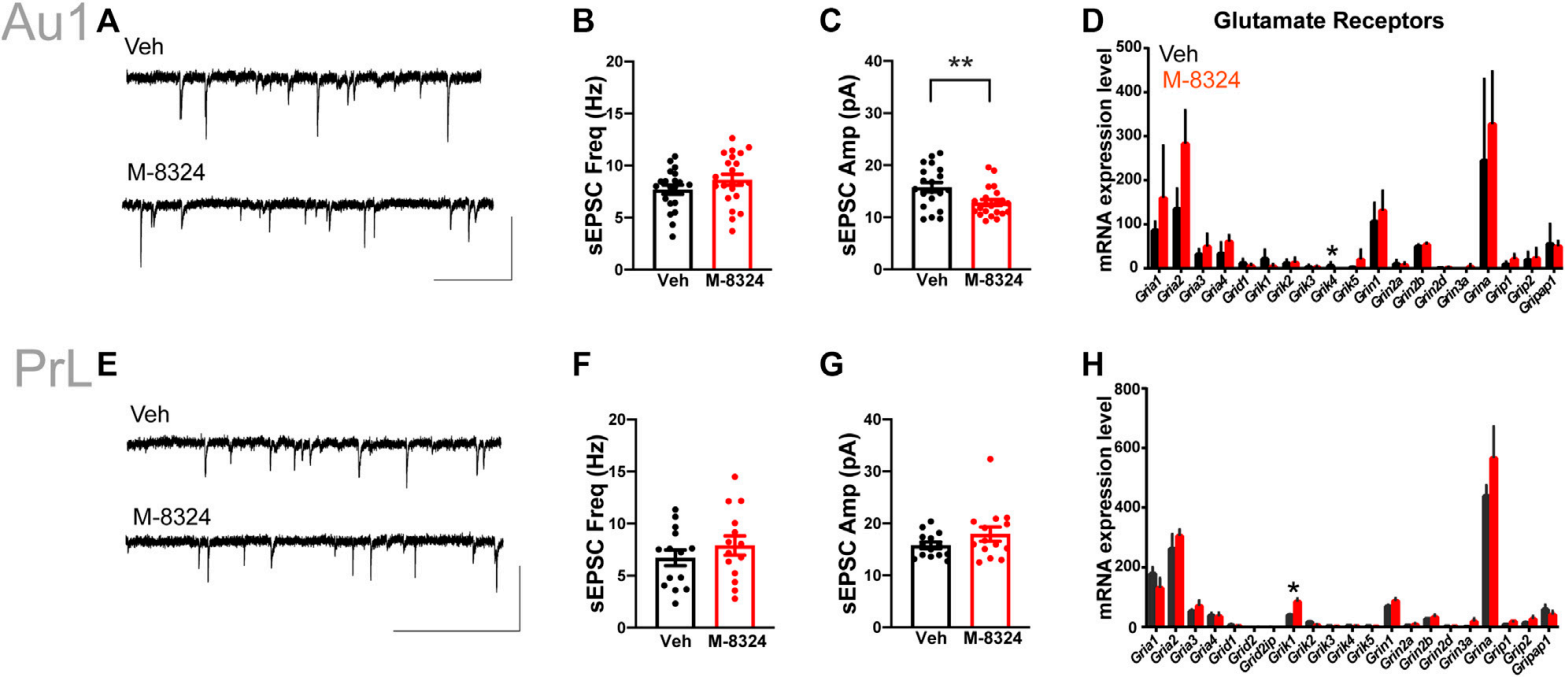
Changes in excitatory synaptic transmission and glutamate receptors in PV-neurons after 7-days M-8324 treatment in Au1 and PrL-PFC. **(A)** Representative traces of sEPSC from Au1 PV-neurons in Veh and M-8324 groups. Scale bars, 50 pA/500 ms. **(B)** Frequency of sEPSC was not different between Veh and M-8324 groups in Au1. **(C)** A significantly lower amplitude of sEPSC in M-8324 group compared to Veh group in Au1. **(E)** Representative traces of sEPSC in PV-neurons in Veh and M-8324 groups in PrL-PFC. Scale bars, 50 pA/500 ms. **(F)** Frequency of sEPSC was not different between Veh and M-8324 group in PrL-PFC. **(G)** Amplitude of sEPSC was not different between Veh and M-8324 group in PrL-PFC. **(D,H)** Expressions of Glutamate receptor subunits in PV-neurons identified in Au1 **(D)** and PrL-PFC **(H)** by RNA-seq. N = 20 cells/3 mice for both groups in Au1, N = 14 cells/3 mice for both groups in PrL-PFC. *p < 0.05. **p < 0.01.

Since M-8324 selectively enhances the NMDAR activity and neuronal activity in the GABAergic neurons, we next wanted to understand whether long-term enhancement may lead to persistent changes in these neurons. To do so, we selected to focus on PV-neurons, one major class of GABAergic neurons in the brain (Kawaguchi and Kubota, 1997; Uematsu et al., 2008; Rudy et al., 2011). Since no direct comparison between PV-neurons in Au1 and PrL-PFC in naïve animals has been done, it is of interest to understand whether distinct electrophysiological properties and functionally relevant genes exist. We recorded from genetically identified PV-neurons in *PV:Cre/Ai9* mice in the whole-cell patch recording mode. The intrinsic excitability was different in that Au1 PV-neurons showed lower excitability than PrL-PFC PV-neurons in the low current injection range (Figure 2A). Spikes evoked by current injection were significantly different: (1) amplitudes were significantly larger in Au1 PV-neurons (Figure 2B), (2) half-width was significantly larger in the Au1 PV-neurons (Figure 2C), (3) rheobase current, a measure of how much current is required to elicit a spike, was significantly larger in Au1 PV-neurons (Figure 2D), and (4) AHP latency was significantly shorter in Au1 PV-neurons (Figure 2E). Differences in the latter two parameters are consistent with the lower excitability in Au1 PV-neurons. We also examined the basic properties of synaptic transmission in PV-neurons in Au1 and PrL-PFC and found no significant difference between them (data not shown), suggesting that they differ only in the properties of spikes/excitability.

To understand whether large molecular differences exist in PV-neurons between Au1 and PrL-PFC, we sequenced PV-neurons (see Methods). A total of 15,345 genes were detected (Supplementary Table S1), which is the largest number of genes reported for PV-neurons at this time. We found a large number of DEGs between Au1 and PrL-PFC (compared with Au1, 909 genes were up-regulated and 752 genes were down-regulated in PrL-PFC) (Supplementary Figure S1A and Supplementary Table S1), and more genes were PrL-PFC unique than Au1 unique (9.9% in PrL-PFC and 5.5% in Au1) (Supplementary Figure S1C). These results suggest that the PV-neuron transcriptomes of Au1 and PrL-PFC are distinct. We first performed GO functional enrichment analysis for the above identified genes. For cellular component, although significantly enriched GO terms in Au1 and PrL-PFC are not the same, they are both mainly related to extracellular components and collagen (Supplementary Figure S2). In PrL-PFC, two GO terms related to plasma membrane are also significantly enriched (Supplementary Figure S2). For molecular functions, protein tyrosine kinase activity and beta-2-microglobulin binding are enriched in Au1 while calcium ion binding and protein-glutamine gamma-glutamyltransferase activity are significantly enriched in PrL-PFC (Supplementary Figure S2). For biological processes, cell adhesion and homophilic cell adhesion via plasma membrane adhesion molecules are the top two enriched genes in PrL-PFC but not Au1 (Supplementary Figure S2). Put together, the main differences between PV-neurons in these two brain regions are plasma membrane and extracellular matrix, and genes related to cell adhesion of PV-neurons appear to be more enriched in PrL-PFC than in Au1.

Ion channels and GABA or glutamate receptors are two main membrane protein classes most relevant to the electrophysiological activity of neurons, so we then asked whether genes encoding them are different between Au1 and PrL-PFC. Consistent with no significant difference in synaptic transmission, both types and expression levels of GABARs and GluRs are not significantly different between Au1 and PrL-PFC, except that GABA_A_ receptor β2 subunit (*Gabrb2*) was significantly higher expressed in PrL-PFC (Supplementary Figure S3A–B and Supplementary Table S2). As for ion channels, although most genes (83.1%) are shared and expressed indifferently, Au1 and PrL-PFC have their own expression profiles (Figure 2F). Specifically, 6 K^+^ genes were unique (*Kcnq1*, *Kcng3*, *Kcng4*, *Kcnk7*, and *Kctd15*) or higher expressed (*Kctd16*) in Au1, and 9 K^+^ subunits were unique (*Kcns1*, *Kcns2*, *Kcng2*, *Kcna5*, and *Kcnk15*) or higher expressed (*Kcnc2*, *Kcnj3*, *Kctd7,* and *Kcnmb3*) in PrL-PFC (Figure 2F, right; Supplementary Table S3). Among these genes, *Kcnc2, Kcnj3,* and *Kctd7* showed the highest expression levels, and may have important contributions to the higher intrinsic excitability of PrL-PFC PV-neurons. In addition, *fibroblast growth factor 12* (*Fgf12*) showed significantly higher expression in PrL-PFC and is known to increase neuronal excitability by enhancing the depolarization shift in Nav1.6 (encoded by *Scn8a*) voltage-dependent fast inactivation (Siekierska et al., 2016). Hence, it may also have a significant contribution to the higher intrinsic excitability in PrL-PFC PV-neurons. In general, types and expression levels of Na^+^ and Ca^2+^ channel subunits exhibited smaller difference than K^+^ channels between Au1 and PrL-PFC, with only *Scn4b, Cacng5,* and *Cacna1e* significantly higher expressed in Au1 (Supplementary Figures S3C–D, S4).

Cell adhesion molecules (CAMs) are also a very large family membrane proteins and play key roles in synapse localization, identity, and functions (Paul et al., 2017; Sudhof, 2017; 2018). Based on the CAMs reported in previous studies (Foldy et al., 2016), we identified a total of 324 genes (Supplementary Table S5). Gene expression pattern of CAMs was different between Au1 and PrL-PFC (Figure 2G, left). Although 75.5% genes are shared, Au1 and PrL-PFC have their own unique CAMs expression profile. In addition, more genes were selectively expressed in PrL-PFC than in Au1 (42 vs. 26) (Figure 2G, right; Supplementary Table S7). There were 7 and 5 CAMs with significantly higher expression in PrL-PFC and Au1, respectively (Figure 2G, right; Supplementary Table S5). Base on sequence homology and receptor-ligand relationships, CAM genes have been divided into 9 main categories (Paul et al., 2017). Au1-specifically and significantly higher expressed CAMs and PrL-specifically and significantly higher expressed CAMs were listed in Fig. S4. The best characterized synaptic CAMs, such as *neurexins*, *neuroligins,* and *cadherins*, were not differentially expressed between Au1 and PrL-PFC. Among these CAMs genes, *neurexophilin-1* (*Nxph1*) stands out because its expression is very high in both Au1 and PrL-PFC, and its expression level was significantly higher in PrL-PFC (Supplementary Figure S4 and Supplementary Table S5). *Nxph1* is an *α-neurexin* ligand with selective expression in subpopulations of inhibitory neurons and plays an instructive role in synaptic short-term plasticity. As reported, cell-specific expression of leucine-rich repeat proteins (LRRs) might contribute to post- and *trans*-synaptic specializations that customize the property of synapse types defined by pre- and post-synaptic neuron identities (Paul et al., 2017). We found that PV-neurons in Au1 and PrL-PFC expressed unique LRRs (e.g., *Lrrc3b* in PrL-PFC, *Lrrc25* in Au1), which may participate in the differential connectivity patterns of PV-neurons in these regions. Protein tyrosine phosphatases are another major synaptic adhesion family, with higher *Ptpn2* expression in PrL-PFC than in Au1. Notably, more clustered protocadherin (*Pcdh*) genes (46.9% in PrL-PFC vs. 29.0% in Au1) were enriched in the PrL-PFC-specific genes (Supplementary Figure S4 and Supplementary Table S5). Taken together, while most CAMs in PV-neurons are shared between Au1 and PrL-PFC, unique expression profiles are identified, with PrL-PFC exhibiting a more complex cell-surface assembly code.

### Minor Impact of 7-Days M-8324 Treatment on Cell Cycle and Apoptosis

One goal of the current study is to evaluate potential excitotoxicity associated with prolonged activation of NMDARs. We thus focused on genes associated with cell cycle and apoptosis pathways. Apoptosis in inhibitory neurons is critically dependent on the intrinsic pathway marked by increased *BCL2-associated X protein* (*Bax*), decreased *B cell leukemia 2* (*Bcl-2*), and cleaved-caspase 3 (Krasnova et al., 2005; Duan et al., 2020). None of these 3 genes was significantly altered after M-8324 treatment in either Au1 or PrL-PFC (Supplementary Figure S5, S6), indicating no significant impact on the apoptosis pathway. For cell cycle pathway, *Cell division cycle* (*Cdc*), *Cyclin dependent kinases* (*CDKs*), and *Cyclin*s are three key regulators (Schafer, 1998) [46]. None of them was significantly altered by M-8324 treatment in PrL-PFC, except that the *CycB* level was slightly higher in the M-8324 treatment group. However, *CDK1* was not affected and cell cycle pathway might not be altered significantly (Supplementary Figure S7). In Au1, *CycA* and *Cdc6* were significantly down-regulated, while *CycB* was significantly up-regulated, but none of *CDKs* was significantly altered with the downstream partner of *Cdc6*: *ORC* slightly increased. Hence, whether cell cycle pathway in Au1 is significantly altered cannot be determined (Supplementary Figure S8). Overall, M-8324 treatment does not have significant impact on pathways related to cell cycle or neuronal death.

### Changes in Intrinsic Excitability and Ion Channel Expression in PV-Neurons After 7-Days M-8324 Treatment.

In PrL-PFC, spike pattern (Figure 3A) and intrinsic excitability (Figure 3B) were not significantly different between PV-neurons from M-8324- and Veh-treated mice. We noticed that this altered excitability occurred mostly at low current injection range (<200 pA). We found significant differences in spike amplitude (Figure 3C), spike trough (the lowest Vm of an AP) (Figure 3E), and magnitude of peak to trough (Figure 3F), but not in the delay to first spike in a spike train (Figure 3D), between PV-neurons from M-8324- and Veh-treated mice. For Au1 PV-neurons treated with M-8324, a significantly higher intrinsic excitability in the low current injection range (Figures 3G,H,J–K), and a significantly smaller delay to first spike were seen (Figure 3I,L), and both were consistent with enhanced excitability.

We then examined whether altered ion channel expression may account for the above electrophysiological differences. For K^+^ channels, compared to PrL-PFC, Au1 has more differentially expressed genes [14 (15.6%) *vs.* 6 (6.7%)], with only two genes (i.e., *kcnn1* and *kcnu1*) shared (Figure 3M). Among the Au1-specific genes, 3 Kv subunits (*Kcns1-3*) and *Kctd7* were up-regulated, and 9 Kv subunits (*Kcna4*, *Kcnb2*, *Kcnd1*, *Kcnf1*, *Kcng1*, *Kcng3*, *Kcng4*, *Kcnk7*, *Kcnk13*) and *Kctd21* were down-regulated (Figure 3M; Supplementary Table S3). Among them, *Kcns3* showed the highest expression with a 4.2-fold upregulation in the Au1 of M-8324 group. In addition, *Kcns1* and *Kcns2* were PrL-PFC-specific and up-regulated in Au1 of M-8324 group. Among the PrL-PFC-specific genes, 2 Kv subunits (*Kcnh7* and *Kcnq2*) and *Kcnn3* were up-regulated, while *Kctd12*, *Kcng2,* and *Kcnj13* were down-regulated (Figure 3M and Supplementary Table S3). Among them, *Kcnh7* and *Kcnq2* showed highest expression. Overall, the above results indicate that certain K-channels related functions are likely altered after M-8324 treatment. As for Nav and Cav channels, two Nav genes (*Scn4b* and *Scn7a*) were specifically down-regulated in Au1 and no differential Nav genes were found in PrL-PFC after long-term M-8324 treatment (Figure 3N and Supplementary Table S4). Ca^2+^ channel subunit *Cacng8* was specifically down-regulated in Au1, while *Cacng3*, *Cacng4,* and *Cacfd1* specifically up-regulated in PrL-PFC (Supplementary Figure S9 and Supplementary Table S4).

In summary, after 7-days M-8324 treatment, K^+^ channel-related genes in Au1 and PrL-PFC have undergone differential changes, with few differentially expressed genes shared and more genes modulated in Au1.

### Impact of 7-Days M-8324 Treatment on Neurotransmission Onto PV-Neurons

**FIGURE 5:**
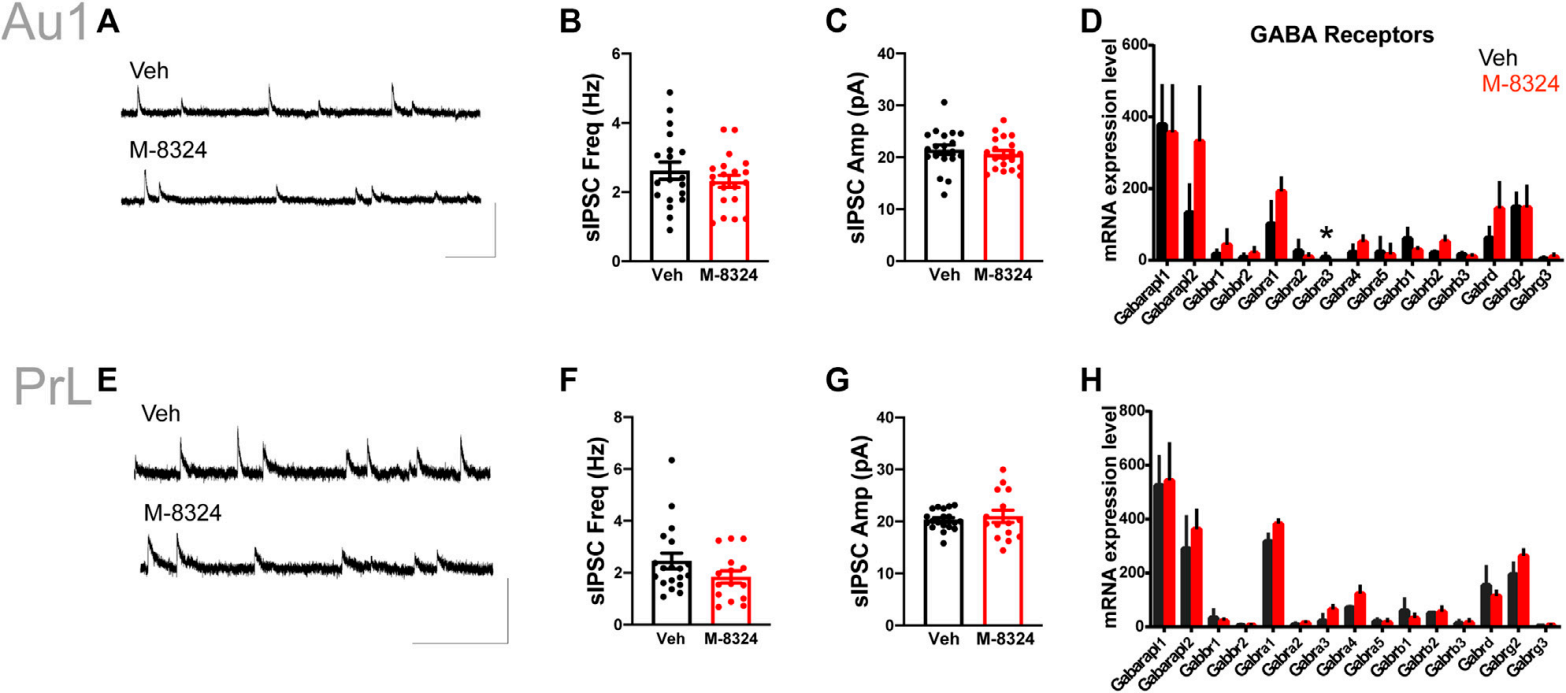
Inhibitory synaptic transmission and expression of GABA receptors in PV-neurons after 7-days M-8324 treatment in Au1 and PrL-PFC. **(A)** Representative traces of sIPSC in PV-neurons in Veh and M-8324 groups in Au1. Scale bars, 50 pA/500 ms. **(B)** Frequency of sIPSC was not different between Veh and M-8324 groups in Au1. **(C)** Amplitude of sIPSC was not different between Veh and M-8324 groups in Au1. **(E)** Representative traces of sIPSC from PV-neurons in Veh and M-8324 groups in PrL-PFC. Scale bars, 50 pA/500 ms. **(F)** Frequency of sIPSC was not different between Veh and M-8324 groups in PrL-PFC. **(G)** Amplitude of sIPSC was not different between Veh and M-8324 groups in PrL-PFC. **(D,H)** Expressions of GABA receptor subunits in PV-neurons identified in Au1 **(D)** and PrL-PFC **(H)** by RNA-seq. N = 18 cells/3 mice for both groups in Au1, N = 19 cells/3 mice for Veh and N = 15 cells/3 mice for M-8324 group in PrL-PFC. *p < 0.05.

No alteration in sEPSCs was seen in PV-neurons except for significantly smaller sEPSC amplitude in Au1 (Figure 4A–C,E–G). Among the detected glutamate receptor subunits and their associated/interacting proteins, only *Grik4* was significantly down-regulated in Au1 and only *Grik1* significantly up-regulated in PrL-PFC in the M-8324 group (Figures 4D,H and Supplementary Table S2). The expression of all NMDA and AMPA receptor subunits did not change significantly in either Au1 or PrL-PFC after M-8324 treatment, including *Grin2a*, the target of M-8324 (Figures 4G,H and Supplementary Table S2). There was no significant alteration in sIPSCs in PV-neurons except a slightly larger sIPSC decay time in PrL-PFC (Supplementary Figure S10; Figures 5A–C,E–G), suggesting minimal changes in GABAergic transmission onto PV-neurons in either region. Consistently, no significant change was found in GABA receptor subunits, except for *Gabra3* being significantly down-regulated in Au1 (Figures 5D,H and Supplementary Table S2).

### Au1 Showed a More PrL-like CAM Profile After 7-Days M-8324 Treatment

**FIGURE 6:**
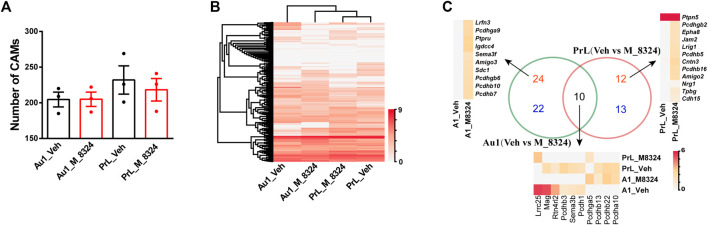
Distinct gene expression patterns of cell adhesion molecules in PV-neurons after 7-days M-8324 treatment. **(A)** Numbers of all detected CAMs were not different between Veh and M-8324 in both Au1 and PrL-PFC. **(B)** Clustering heatmap of expression pattern of all detected CAMs in PV-neurons after M-8324 treatment in Au1 and PrL-PFC. **(C)** A Venn diagram showing difference and similarity of differential CAMs after M-8324 treatment between Au1 and PrL-PFC. Red numbers indicate the number of up-regulated genes, blue the down-regulated genes, and black the differential genes shared. Heatmap and names of the up-regulated genes with expression value ≥ 4.0 in either group in Au1 (top 10) and all up-regulated genes in PrL-PFC were showed besides the Venn diagram. Heatmap and names of all differential genes shared by Au1 and PrL-PFC were shown under the Venn diagram. Standardized method of the heatmaps: log(value +1).

We also analyzed changes in CAMs after 7-days M-8324 treatment in Au1 and PrL-PFC. First, M-8324 treatment did not significantly affect the total number of CAMs expressed in either Au1 or PrL-PFC (Figure 6A). Interestingly, by using hierarchical cluster analysis we found a close match of CAMs expression profiles in Au1 and PrL-PFC after M-8324 treatment. Au1-M-8324 and PrL-PFC groups were clustered into one category while the Au1-Veh group was clustered into another category, suggesting that Au1 exhibits a more PrL-like CAM profile after M-8324 treatment (Figure 6B and Supplementary Table S5). We focused on two groups of CAMs: one with higher expression in PrL-PFC in naïve mice and significantly up-regulated in Au1 of M-8324 treated mice (termed “high-up”), and another with lower expression in PrL-PFC in naïve mice and significantly down-regulated in Au1 of M-8324 treated mice (termed “low-down”). Do the above altered genes belong to any specific functional categories? We found that the “high-up” CAMs belong to “neuronal-IgCAMs,” “LRR + Slitrk + Elfn + Lphn,” “Pcdhs,” “Protein Tyrosine phosphatases,” “Semaphorin + Plexin,” and other undefined categories, with the largest number of genes belong to Pcdhs (Supplementary Figure S11). The “low-down” CAMs belong to “neuronal-IgCAMs,” “LRR + Slitrk + Elfn + Lphn,” “Pcdhs,” “Eph + EphR,” “Netrin-Unc5-Slit-Robo,” and other undefined categories. Notably, 83.3% of the above altered genes was either up-regulated from zero or down-regulated to zero. Among them, *Lrrc3b*, *Tlr1*, and *Lrrc25* stand out because of their relatively high expression level and level of modulation (Supplementary Figure S11). The best characterized synaptic CAMs, such as *neurexins*, *neuroligins* and *cadherins*, were absent.

We then analyzed the similarities and differences of the differentially expressed CAMs after M-8324 treatment in Au1 and PrL-PFC. First, more CAMs were differentially expressed only in Au1 than in PrL-PFC (46 DEGs in Au1, 56.8% of the total differential CAMs vs. 25 DEGs in PrL-PFC, 30.9% of the differential CAMs) (Figure 6C and Supplementary Table S5). The Au1-specific and PrL-PFC-specific CAMs belong to 8 categories, excluding the best characterized synaptic CAMs (*neurexins*, *neuroligins,* and classical *cadherins*) (Supplementary Table S5). Nevertheless, other major synaptic adhesion families (e.g., protocadherins, protein tyrosine phosphatases, leucine-rich repeat proteins, and Slitrks) were differentially expressed in Au1 and PrL-PFC. There were 18 differentially expressed Clusted pcdhs in Au1 and 10 in PrL-PFC (Supplementary Table S5). Seven (*Igdcc4, Sema3e, Amigo3, Sema3f, Iglon5, Kit,* and *Icam1*) and two (*Amigo2* and *Jam2*) neuronal-IgCAMs were significantly altered in Au1 and PrL-PFC, respectively (Supplementary Table S5). Four (*Ptpre*, *Ptpn2*, *Ptpru,* and *Ptprc*) and one (*Ptpn2*) Protein Tyrosine phosphatase were significantly altered in Au1 and PrL-PFC, respectively (Supplementary Table S5). Four (*Lrrc3b*, *Lrfn3*, *Lrrtm1,* and *Lrrn2*) and 2 (*Lrig1* and *Efna1*) “LRR + Slitrk + Elfn + Lphn” were significantly changed in Au1 and PrL-PFC, respectively (Supplementary Table S5). These results indicate that after M-8324 treatment, a significantly higher number of CAM genes were modulated in Au1 than in PrL-PFC, and Au1 and PrL-PFC exhibit unique CAM modulation patterns.

### Distinct Up-Stream and GO Enrichment in Au1 and PrL-PFC Following 7-Days M-8324 Treatment

**FIGURE 7:**
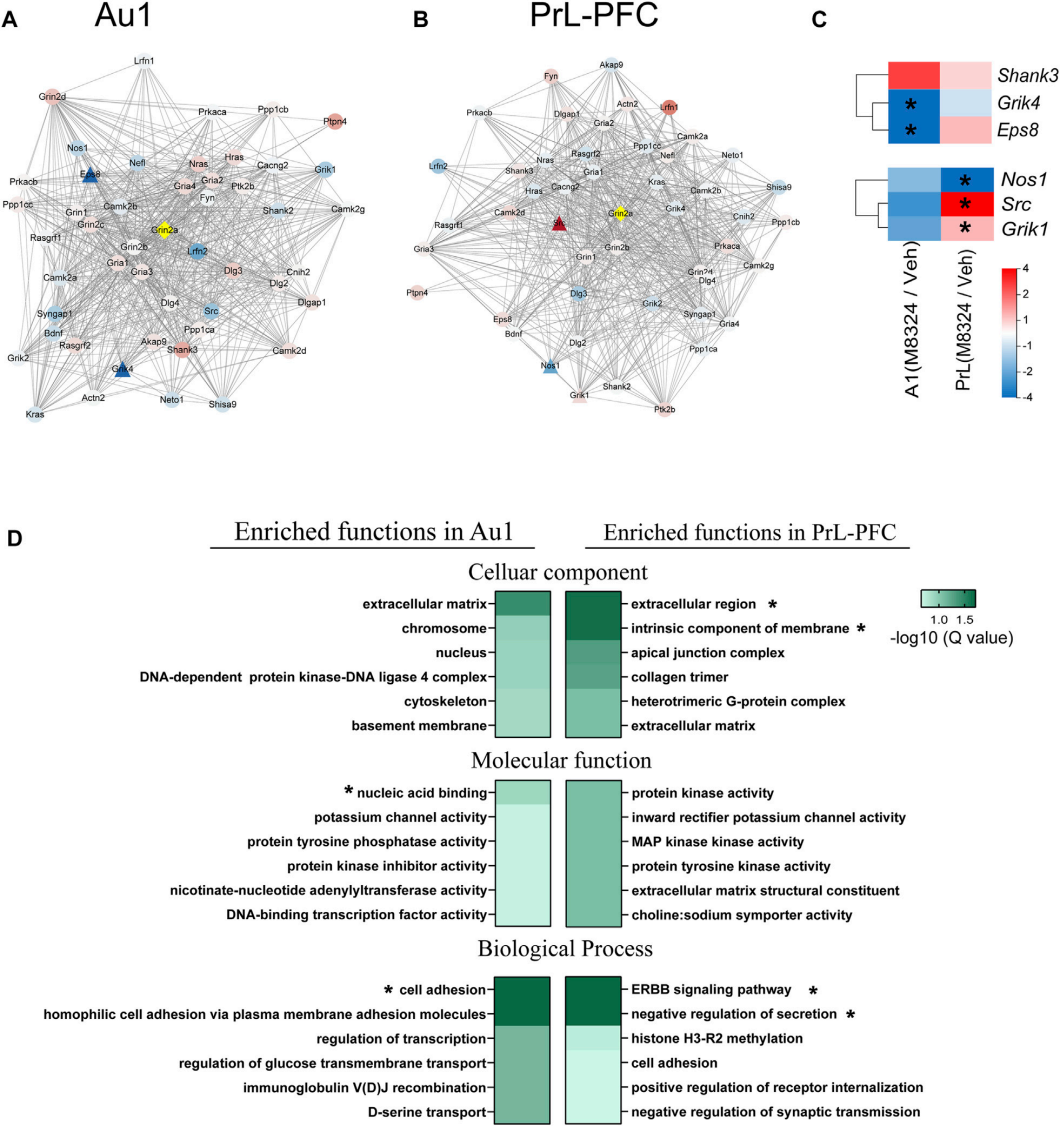
GO functional enrichment and protein-protein interaction of *Grin2a* in Au1 and PrL-PFC after 7-days M-8324 treatment. **(A–B)** Protein-protein interaction (PPI) network of *Grin2a* with the first shell of 50 nodes shown. The active interaction sources came from textmining, experiments, databases, co-expression, neighborhood, gene fusion or co-occurrence. Gene expression difference in *Grin2a*-interacting proteins after M-8324 treatment in Au1 and PrL-PFC were shown in **(A,B)**, respectively. **(C)** Heatmaps of the significantly changed nodes from data in **(A,B)**. For panels **A**–**C**, fold changes are shown in color gradient, the redder the color the greater the up-regulation, and the bluer the color the greater the down-regulation. Significantly differentially expressed genes between Veh and M-8324 groups are represented by triangles while the rest by circles. **(D)** Biological functions of all up-regulated DEGs using a GO enrichment analysis, with top 6 enriched term shown. Colors represent the enriched Q-value, the deeper the color the smaller the Q-values. * *Q value* < 0.05.

Since M-8324 is a NMDAR-PAM targeting GluN2A (*Grin2a*)-containing NMDARs, we focused on molecules that interact with *Grin2a*. Protein-protein interaction (PPI) network analysis of *Grin2a* was used to reveal affected genes immediately downstream of *Grin2a*, a starting point for subsequent functional changes. First, compared to PrL-PFC, M-8324 may have a greater impact on *Grin2a*-interacting genes in Au1 based on significantly larger fold of change in Au1 (Figures 7A,B). Second, the *Grin2a*-interacting genes that are significantly differentially expressed after M-8324 treatment were non-overlapping between Au1 and PrL-PFC (Figure 7C). *Eps8* and *Grik4* were significantly down-regulated after M-8324 treatment in Au1 (Figure 7C). *Shank3* showed the largest up-regulation, although not significantly altered in Au1. *Src* and *Grik1* were significantly up-regulated after M-8324 treatment in PrL-PFC. In addition, *Nos1* was significantly down-regulated (Figure 7C). Among these altered genes, *Eps8*, *Src,* and *Grik1* show opposite directions between Au1 and PrL-PFC, and the rest were altered in the same direction but with different fold of change, suggesting the different primary regulations might be activated after M-8324 treatment.

After 7-days M-8324 treatment, more DEGs were found in Au1 (705 up-regulated and 937 down-regulated) than in PrL-PFC (402 up-regulated and 621 down-regulated) (Supplementary Figure S1A). Most of these genes are unique to either Au1 or PrL-PFC, with small overlap (only 38 up-regulated and 90 down-regulated) (Supplementary Figure S1B), suggesting that M-8324 treatment induces distinct and more changes in the transcriptome of Au1 comparing to PrL-PFC. Since the GO function enrichment analysis of DEGs allowed us to gain a comprehensive understanding of the main affected functions by M-8324 treatment, we performed such analysis. We found that Au1 and PrL-PFC were significantly enriched in different GO terms and more GO terms were significantly enriched for down-regulated genes than up-regulated genes. More specifically, for up-regulated genes, only 3 terms (nucleic acid binding, cell adhesion, and homophilic cell adhesion via plasma membrane adhesion molecules) were significantly enriched in Au1 and 4 terms (extracellular region, intrinsic component of membrane, ERBB signaling pathway, and negative regulation of secretion) were significantly enriched in PrL-PFC (Figure 7D). As many as 18 terms and 10 terms were significantly enriched for down-regulated genes in Au1 and PrL-PFC, respectively (Supplementary Table S6). The enriched 18 GO terms could be roughly divided into three functional categories, covering cell and synaptic adhesion, cell projection, and signaling transduction in Au1. In PrL-PFC, the 10 terms could be roughly divided into membrane and extracellular matrix, protein and ion binding, and cell adhesion. Up-regulated genes annotated to cell adhesion were more enriched in Au1 than in PrL-PFC after M-8324 treatment, and genes being modulated in these two brain regions are quite different.

In summary, after long-term enhancing the activity of NMDARs by M-8324, different primary genes and GO terms were modulated in Au1 vs. PrL-PFC.

## Discussion

How to enhance the function of inhibitory neurons in a disease setting holds great therapeutic potential, but this needs to be thoroughly examined especially for its long-term impact. In this work, we analyzed in wt mice the long-term impact of infusing a NMDAR-PAM M-8324 which selectively enhances the activity of inhibitory neurons. We found (1) electrophysiological properties are largely unaffected in inhibitory neurons; (2) alterations in *vivo* spiking are similar to the acute effects of M-8324 and are largely reversible upon M-8324 washout; (3) differences in the mRNA expression exist between Au1 and PrL-PFC in naïve mice, especially those genes encoding CAMs and K^+^ channel subunits; (4) differentially altered transcriptional profiles in the Au1 and PrL-PFC, and with more alterations in the former and larger changes in genes encoding CAMs and K^+^ channel subunits. We cannot distinguish between direct and indirect effects of M-8324 for the above changes, and some of the identified alterations may be compensatory in nature. Our previous works (Hardingham and Bading, 2010; Yao et al., 2018; Deng et al., 2020; Ge et al., 2020) indicate that M-8324 directly affects the inhibitory neurons and indirectly affects excitatory neuron activity/functions which may also contribute to the above changes.

### Key Electrophysiological Parameters Are Minimally Altered by 7-Days M-8324 Treatments

Our *in vivo* analysis showed that long-term impacts of M-8324 on *in vivo* neural activity and E/I balance are similar to the acute effect, and these effects are reversed after drug washout, suggesting a lack of persistent impact of M-8324 on these parameters. This reversibility is consistent with the finding of Rogers et al. (2021) on the reversible changes in the activity of both excitatory and inhibitory neurons after repeated enhancing the activity of inhibitory neurons in the hippocampus using DREADD. Previous studies suggest that long-lasting alterations in the activity of inhibition of neurons usually are associated with synaptic and homeostatic modifications (Tsodyks et al., 1997; Mitra et al., 2011; Turrigiano, 2011; Rogers et al., 2021). However, our *in vitro* analysis of properties of action potentials, glutamatergic and GABAergic synaptic transmission, and excitability in genetically identified PV-neurons indicate minimal changes in these parameters after M-8324 treatment, and hence limited synaptic and homeostatic modifications. These findings are supported by our RNA-seq finding of minimal alterations in the types and expression levels of GluRs, GABARs, Na^+^ channels, and Ca^2+^ channels subunits.

The impacts of M-8324 on spontaneous activity in Au1 are less affected than evoked activity for the inhibitory neurons. This is the opposite of the acute effect in Au1 and suggests that certain compensatory mechanisms might be in place to exhibit more robust impact on evoked responses following the prolonged elevated inhibitory responses. On the other hand, changes in PrL-PFC towards increased inhibition and decreased excitation result in a significantly reduced E/I ratio, similar to the acute effect. This suggests differential responses and likely distinct plasticity brought upon by NMDAR-PAM treatment in the sensory cortex vs. PFC where the latter appears to be more plastic.

### Impacts on K^+^ Channels by 7-Days M-8324 Treatments

Our in-depth transcriptome analysis on genes encoding K^+^ channels showed (1) large differences between Au1 and PrL-PFC in naïve mice; (2) differentially altered gene expression patterns in the Au1 and PrL-PFC after M-8324 treatment. This brain region-specific gene expression pattern in PV-neurons suggest that their shared molecules are required for core neuronal properties whereas brain region-specific molecules may enable functional specialization for the corresponding regions. In addition, since a tight correlation was found between expression of principal subunits and their matching auxiliary subunits (Paul et al., 2017), our results may imply a brain region-specific assembly of functional channel complex associated with K^+^ channels in PV-neurons. Consistent with enrichment of Na^+^ and K^+^ channels in FS/PV-neurons to maintain their FS properties (Hu et al., 2014; Foldy et al., 2016), we found high expression of K^+^ and Na^+^ channel genes in PV-neurons, especially of *Kcnc1* (encodes Kv 3.1) and *Kcnc2* (encodes Kv 3.2) which are the hallmarks in FS/PV-neurons (Hu et al., 2014). *Kcns3* encoding the Kv 9.3 potassium channel α-subunit is selectively expressed in PV-neurons in human PFC (Georgiev et al., 2012), and Kv9.3 subunits contribute to the precise detection of coincident excitatory synaptic inputs to parvalbumin such as during γ-oscillations (Georgiev et al., 2014). Higher *Kcns3* expression after M-8324 treatment is consistent with higher power of gamma oscillations. Interestingly, lower expression of *Kcns3* was found in PV-neurons in the PFC in schizophrenia patients (Georgiev et al., 2014). *Kcns2* (encoding Kv 9.2) forms functional heterotetrameric channels with KCNB1 and KCNB2 to modulate the delayed rectifier voltage-gated K^+^ channel activation and deactivation rates of KCNB1 and KCNB2. We found that *Kcns1* and *Kcns2* were specifically expressed in PrL-PFC and upregulated after M-8324 treatment in Au1, and it may thus contribute to the higher intrinsic excitability in PrL-PFC in naïve mice and in Au1 after M-8324 treatment. Földy (Foldy et al., 2016) found that AP frequency, threshold, symmetry, and afterhyperpolarization were positively correlated, while AP peak-to-trough and width were inversely correlated with the high expression of Na^+^ and K^+^ channels (such as *Kcnc1* [Kv3.1], *Kcnc2* [Kv3.2], and *Kcng4* [Kv6.4]). We found that *Kcnc2* has the highest expression among K^+^ channel genes in PrL-PFC and its expression was significantly higher than that in Au1, which may underlie the smaller AP half-width in PrL-PFC. However, *Kcng4* is Au1-specific, which is not consistent with the lower AP frequency and higher AP half-width found in Au1. It is possible that the exact electrophysiological properties are the result of polygenic effects, which need to be further examined.

### CAMs in PV-Neurons and Their Modulation by 7-Days M-8324

CAMs initiate the formation of synapses, hold the pre- and postsynaptic sides together, coordinate the precise alignment of pre- and postsynaptic sides, and enable short- and long-term synaptic plasticity (Gerrow and El-Husseini, 2006; Dalva et al., 2007; Sytnyk et al., 2017; Sudhof, 2018). Some adhesion proteins such as N-Cadherin and Desmoglein were found to coimmunoprecipitate with NMDARs, which makes it tempting to speculate that this GluR-cell-adhesion protein complex may provide multiple *trans*-synaptic signaling pathways, whereby adhesion-mediated signaling is coupled to transmitter signaling mechanisms (Husi et al., 2000). Differential expression patterns of CAMs suggest different capacity for cell-surface assemblies which may play key roles in shaping neuronal connectivity and plasticity (Paul et al., 2017). Consistent with high numbers and diverse CAM expression in FS/PV neurons (Foldy et al., 2016), we also found abundant CAM expression in PV-neurons in both Au1 and PrL-PFC. Moreover, about 25% of CAM genes expressed in PV-neurons are Au1 or PrL-PFC unique, with a larger number in PrL-PFC, suggesting potentially higher plasticity or more complex connectivity in PrL-PFC. Previous studies showed FS neurons express significantly higher numbers of CAMs than excitatory neurons (Foldy et al., 2016), with CAM genes uniquely expressed in FS neurons belong to all the categories (Paul et al., 2017). We found distinct CAM expression in PV-neurons in Au1 and PrL-PFC, and PrL-PFC-specific CAMs include those known to be FS neuron-specific and shared by FS and excitatory neurons. In addition, all CAMs up-modulated by M-8324 in Au1 include both FS neuron-specific and FS/excitatory neurons shared genes, suggesting that the regulatory genes underlying CAM expression in Au1 to resemble that of PrL-PFC after long term M-8324 treatment are not unique to FS neurons. Notably, the best characterized cell adhesion molecules, such as *neurexins* (Dean and Dresbach, 2006; Sudhof, 2017) and their ligands *neuroligins* (Dean and Dresbach, 2006), are not brain regions-specific or altered by M-8324 treatment.

We noticed that *Pcdhs* accounted for 44.9% of the PrL-specific and higher expressed CAMs. The *Pcdhs* make up the most diverse group within the cadherin superfamily including ∼60 proteins encoded by the tandem *Pcdha*, *Pcdhb*, and *Pcdhg* gene clusters and another ∼10 non-clustered *Pcdhs*. The *Pcdhs* are expressed predominantly in the nervous system, especially on the synaptic membrane (Sano et al., 1993; Kim et al., 2007). Unlike classical cadherins which typically mediate strong cell adhesion, the impacts of *Pcdhs* on adhesion remains unclear. For example, *Pcdhs* promote moderate association with cell membrane (Schreiner and Weiner, 2010; Molumby et al., 2016; Tarusawa et al., 2016), but may also promote anti-adhesive functions to avoid interaction with membranes (Lefebvre et al., 2012; Kostadinov and Sanes, 2015; Meguro et al., 2015). This combination of pro-adhesion and anti-adhesion likely specifies synaptic connections which are fundamental for proper functioning of neural circuit. *Pcdhs* were more likely to function as a mediator of cell-cell adhesion or a regulator of other signaling molecules (Kim et al., 2011), for example, the PCDH8-protein kinase 2 (TAO2)-p38 MAPK pathway promotes endocytosis of N-cadherin, and subsequently regulates spine dynamics and maintains the shape and density of spines (Yasuda et al., 2007). The uniquely expressed clustered *Pcdhs* in Au1 and PrL-PFC include *Pcdha*, *Pcdhb,* and *Pcdhg* gene clusters, with more were found in PrL-PFC than in Au1. In addition, 2 non-clustered *Pcdhs* (*Pcdh18* and *Pcdh11x*) were uniquely expressed in PrL-PFC but not in Au1. These results suggest more complex connectivity in PrL-PFC since larger number of *Pcdhs* likely provide a larger repertoire of cell surface recognition capacity capable of producing a barcode-like identity for neurons. Similar region-specific expressions of non-clustered *Pcdhs* have also been observed in cortical area during early postnatal development and in caudate putaman and/or hippocampal formation in mature brains (Kim et al., 2007; Hertel et al., 2008; Kim et al., 2010). Interestingly, after long term M-8324 treatment, more *Pcdhs* were significantly modulated in Au1 than in PrL-PFC. Among them, *Pcdh8* was known to antagonize adhesion mediated by C-cadherin and induce internalization of N-cadherin at synapses in response to increased neural activity (Chen and Gumbiner, 2006; Yasuda et al., 2007). *Pcdh8* showed higher expression in PrL-PFC than in Au1, with expression reduced after long-term M-8324 treatment, suggesting a higher adhesion or formation/stabilization of new synaptic connections in PrL-PFC after long -term M-8324 treatment.

A few other differentially expressed CAMs deserve discussion, such as *Nxph1, Ptpn5,* and *contactin3,* based on their contribution to plasticity*. Nxph1* is an α-neurexin–specific ligand only expressed in the inhibitory neurons and known to play an instructive role in GABA_B_R-dependent synaptic short-term plasticity (Born et al., 2014). We found high expression of *Nxph1* in both Au1 and PrL-PFC and higher in PrL-PFC than in Au1. The expression of *Ptpn5* and *contactin3* were significantly increased after M-8324 treatment in PrL-PFC. *Ptpn5* dephosphorylates GluRs and mediates protein binding and receptor trafficking which ultimately control synaptic transmission and plasticity (Won and Roche, 2021). In Fragile X syndrome and schizophrenia, protein levels of STEP encoding by *Ptpn5* are significantly increased (Darnell et al., 2011). *Contactin3* was suggested to participate in the outgrowth and guidance of axons and dendrites, and associated with autism spectrum disorder (Yoshihara et al., 1995; Hussman et al., 2011). Since *neurexins* and *neuroligins* play important roles in synapse formation and synaptic functions, that they are not significantly altered by M-8324 suggests that these synapse-related functions are minimally affected by M-8324, consistent with our other findings. Put together, our results provide potentially important molecular insight into a deeper understanding of the functions of PV-neurons in different brain regions and the contribution of NMDAR to these functions.

### Distinct Primary Signaling Pathways Associated With NMDAR Modulation in Au1 and PrL-PFC

Distinct signaling pathways might be engaged by M-8324 treatment in Au1 and PrL-PFC, as we found pathways more depending on *Eps8-*related in Au1 or *Src*-related signaling in PrL-PFC. *Eps8* is localized to postsynaptic structures as a part of the NMDAR complex (Offenhauser et al., 2006), and it acts as a signaling adapter to control various cellular processes by regulating actin cytoskeleton. *Eps8-*null neurons are resistant to the actin-remodeling activities of NMDAR (Offenhauser et al., 2006). *Eps8* was significantly down-regulated in Au1 after long-term M-8324 treatment, but a trend towards up-regulation in PrL-PFC. In addition, *Shank3* is also a part of NMDAR complex which connects neurotransmitter receptors, ion channels, and other membrane proteins to the actin cytoskeleton (Monteiro and Feng, 2017). A large up-regulation in *Shank3* after M-8324 long-term treatment was seen in Au1 after long-term M-8324 treatment. It thus appears that as part of the NMDAR complex and acting on actin cytoskeleton, *shank3* and *Eps8* may coordinate with each other to mediate the impact of long-term M-8324 treatment on modulating PV-neuron activity in Au1. On the other hand, *Src* which encodes tyrosine-protein kinase was significantly up-regulated in PrL-PFC but towards down-regulation in Au1 after long-term M-8324 treatment. Proteins encoded by *Src* was coimmunoprecipitate with NMDARs (Husi et al., 2000). Thus, *Grin2a* and *Src* are likely associated with each other both structurally and functionally. *Src* plays an important role in regulating cytoskeletal organization (Roskoski, 2004) and in activating other protein tyrosine kinase families participate in a diverse spectrum of biological activities including gene transcription, cell adhesion, and immune response. In summary, these distinct up-stream mechanisms engaged by NMDAR modulation in Au1 and PrL-PFC are consistent with the distinct gene GO enrichment functions seen in these two brain regions.

In summary, our profiling of PV-neurons after long-term treatment with a NMDAR-PAM M-8324 has revealed small alterations in synaptic transmission and excitability, indicating these properties are largely unaffected by enhanced NMDAR activation. In contrast, significant changes in CAMs and K^+^ channels genes occur in a brain region specific manner which highlights potential influences of NMDARs on cell adhesion and K^+^ channel functions in these neurons which has been less appreciated previously.

## Data Availability

The datasets presented in this study can be found in online repositories. The names of the repository/repositories and accession number(s) can be found in the article/Supplementary Material.
