# Supplementary figures and images for: Long-Term Enhancement of NMDA Receptor Function in Inhibitory Neurons Preferentially Modulates Potassium Channels and Cell Adhesion Molecules
Source: Front Pharmacol. 2022 Jan 4;12:796179. doi: 10.3389/fphar.2021.796179 (PMC8764260; doi:10.3389/fphar.2021.796179)

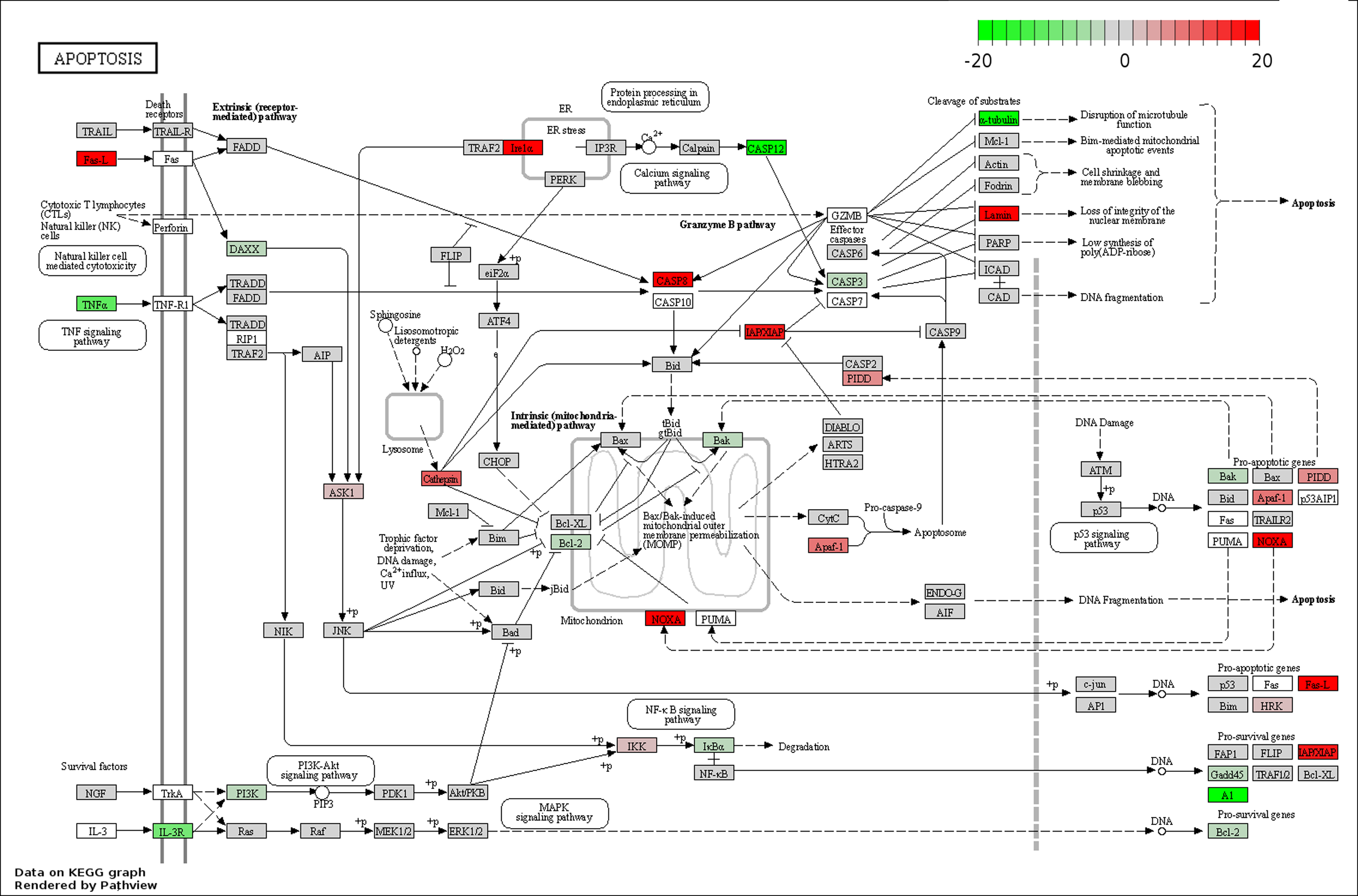

Supplement: Supplementary file 4 [file Image6.TIF]

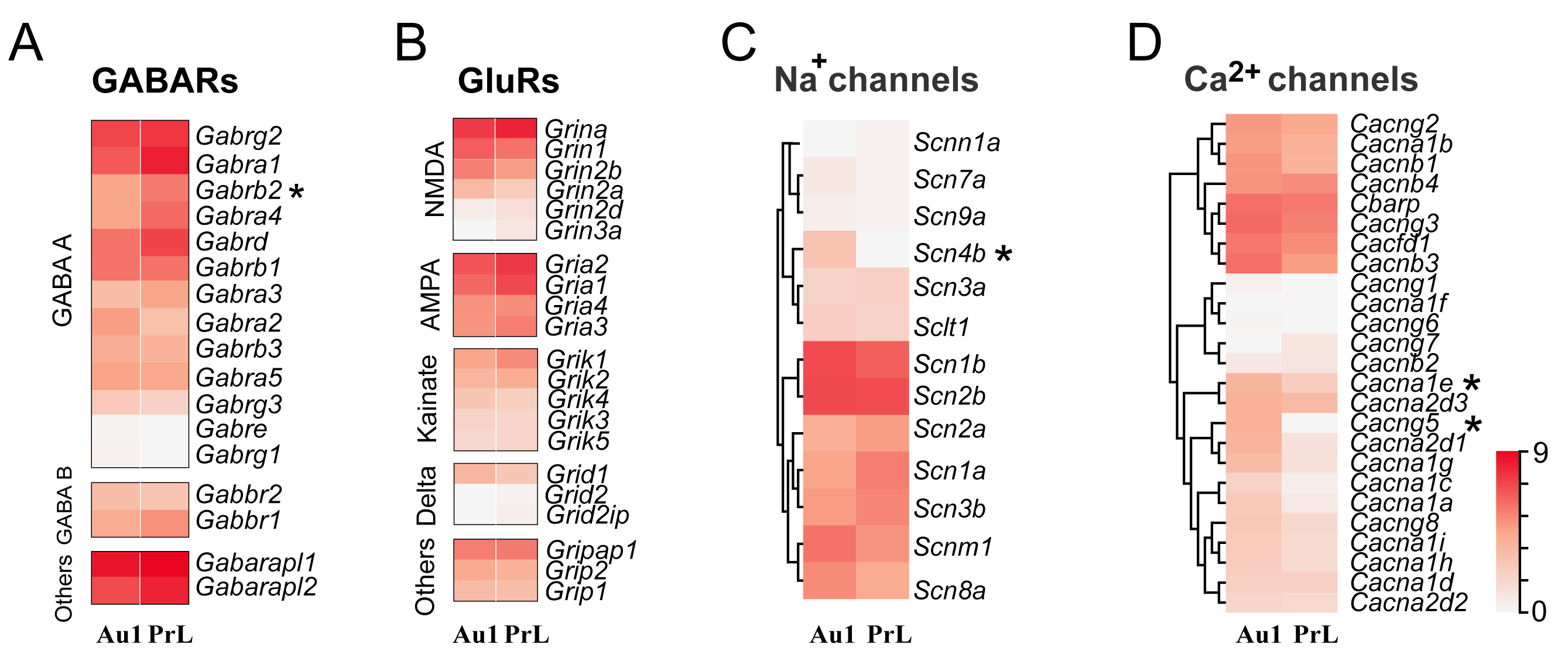

Supplement: Supplementary file 5 [file Image3.TIF]

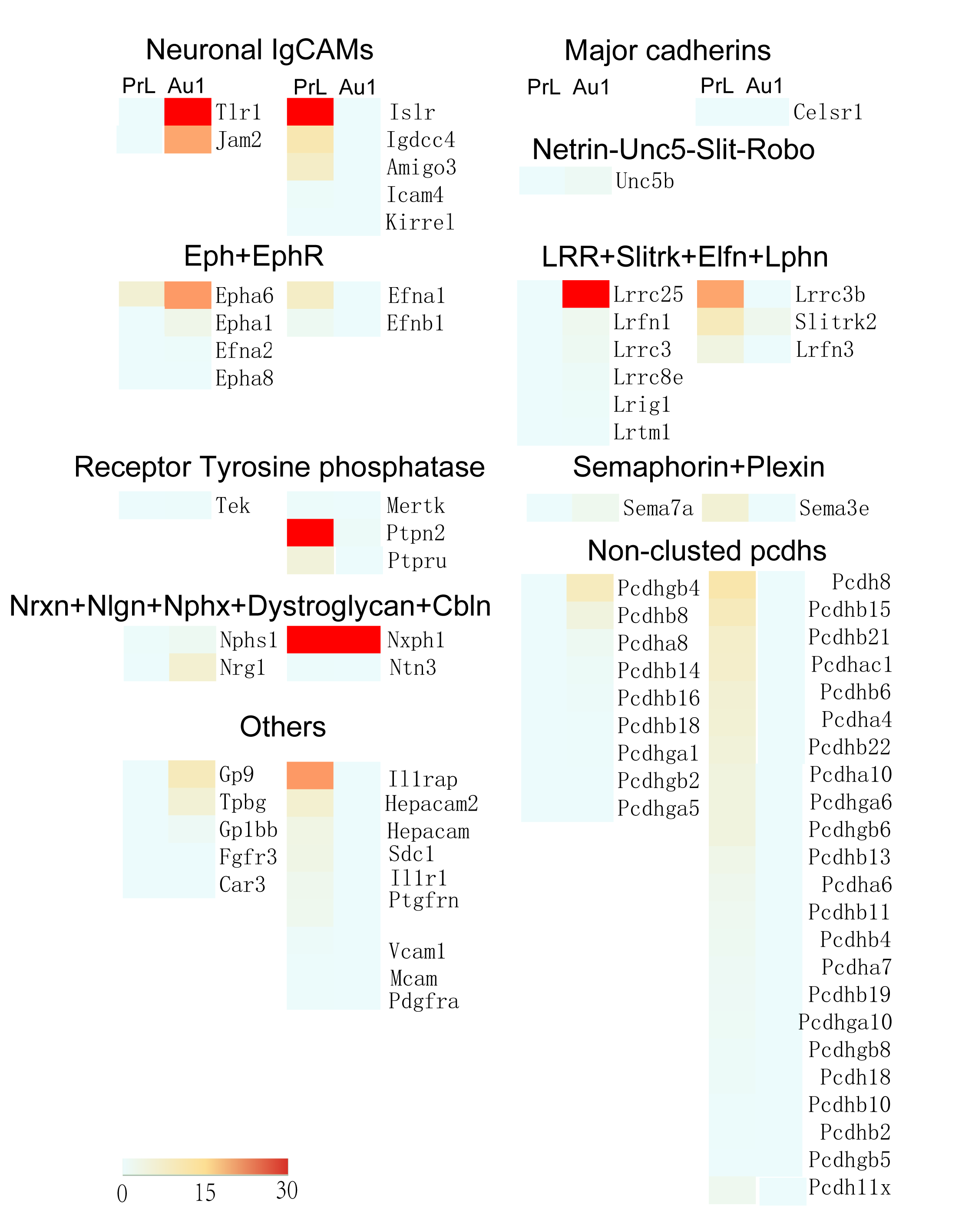

Supplement: Supplementary file 6 [file Image4.TIF]

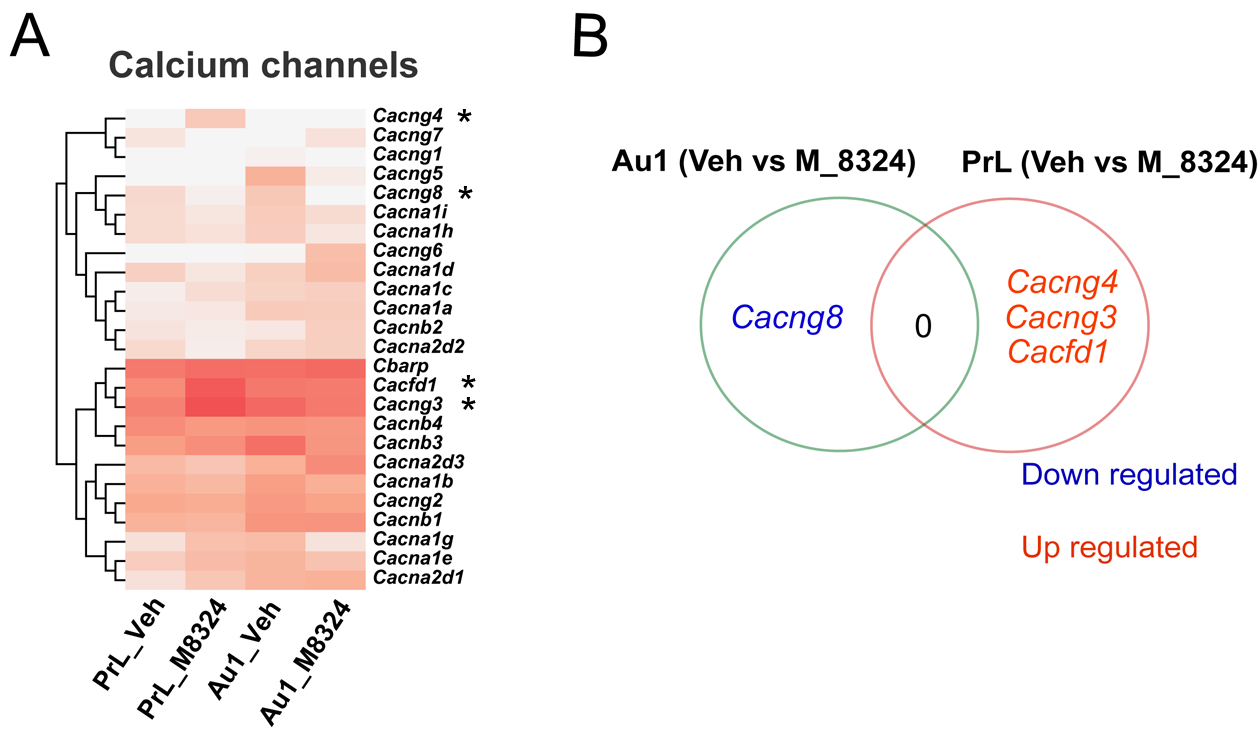

Supplement: Supplementary file 7 [file Image9.TIF]

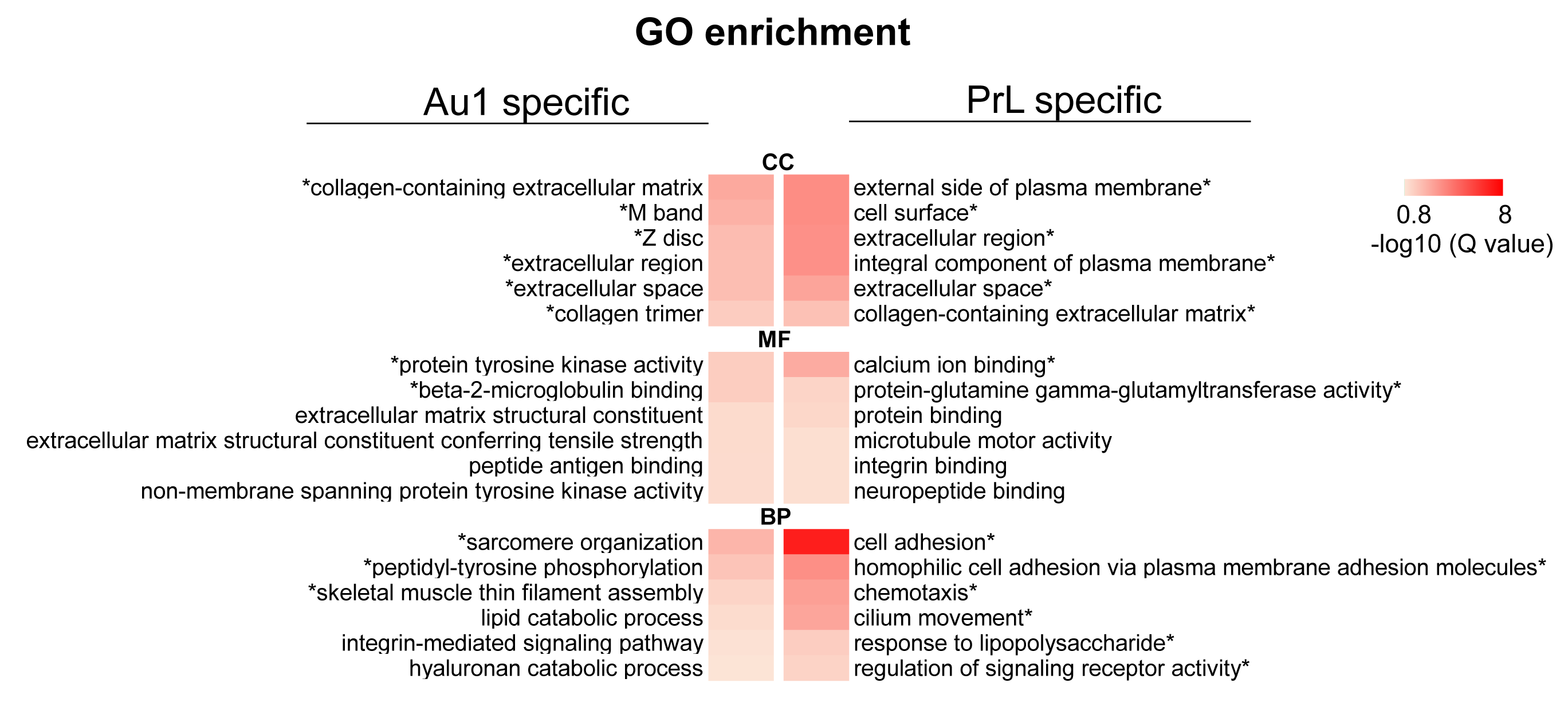

Supplement: Supplementary file 9 [file Image2.TIF]

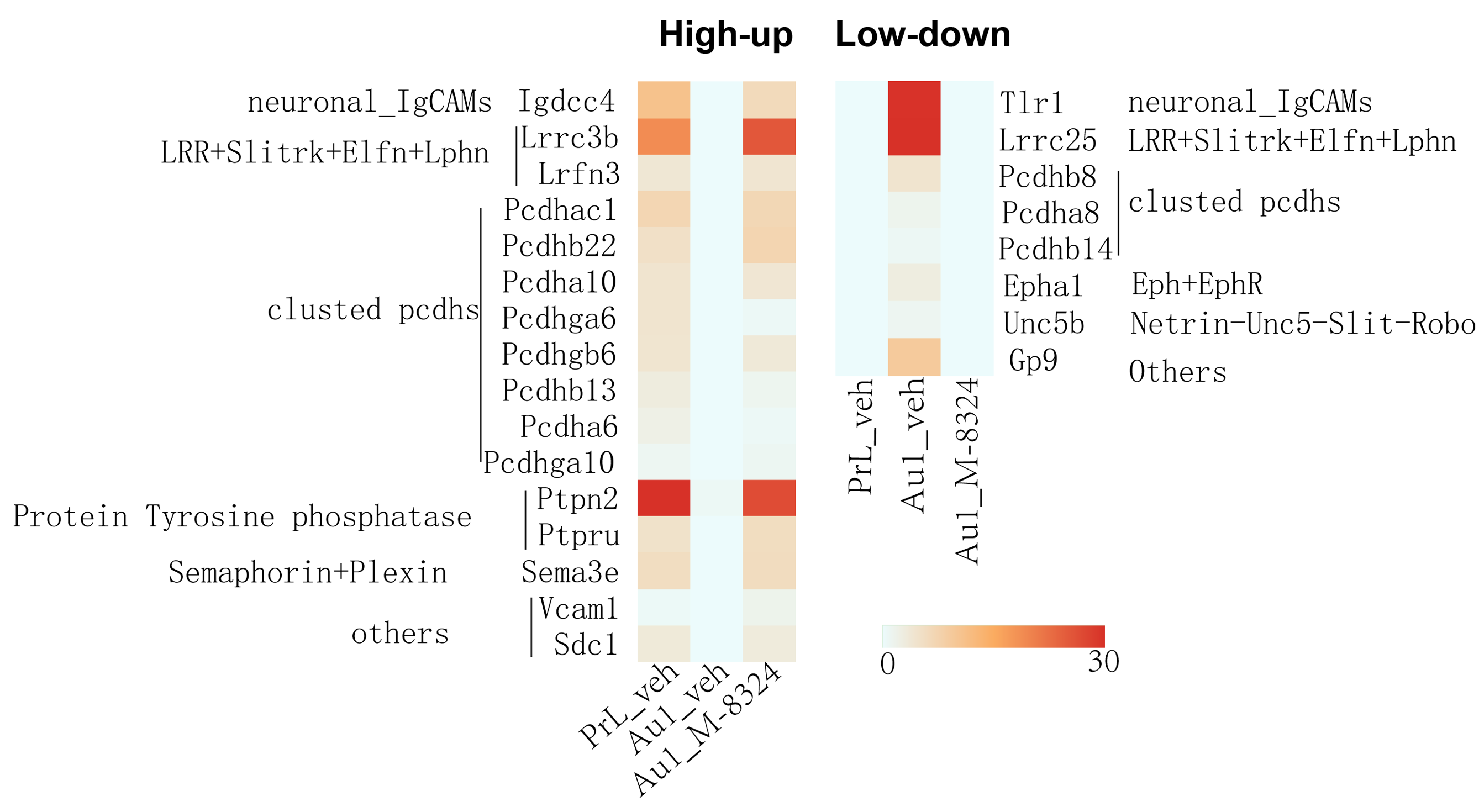

Supplement: Supplementary file 10 [file Image11.TIF]

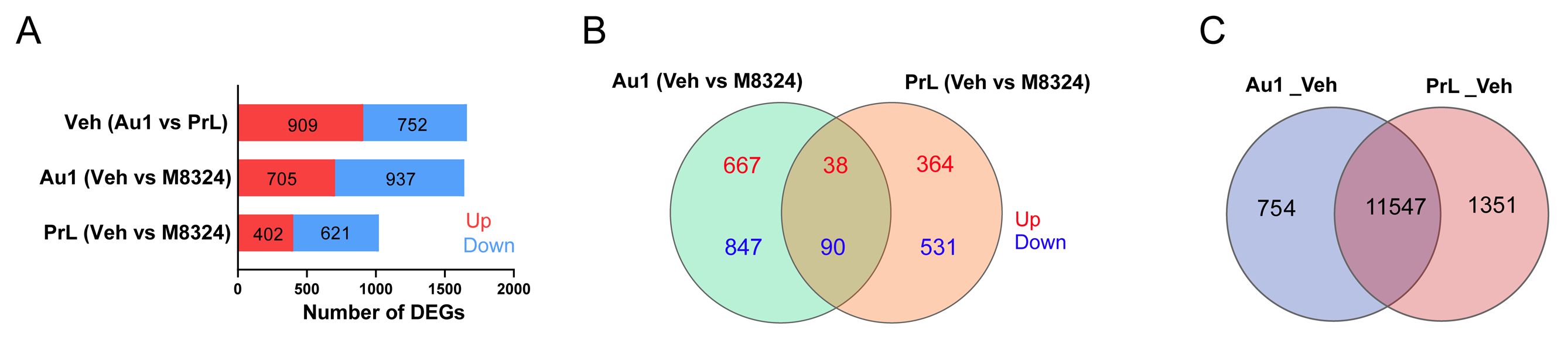

Supplement: Supplementary file 12 [file Image1.TIF]

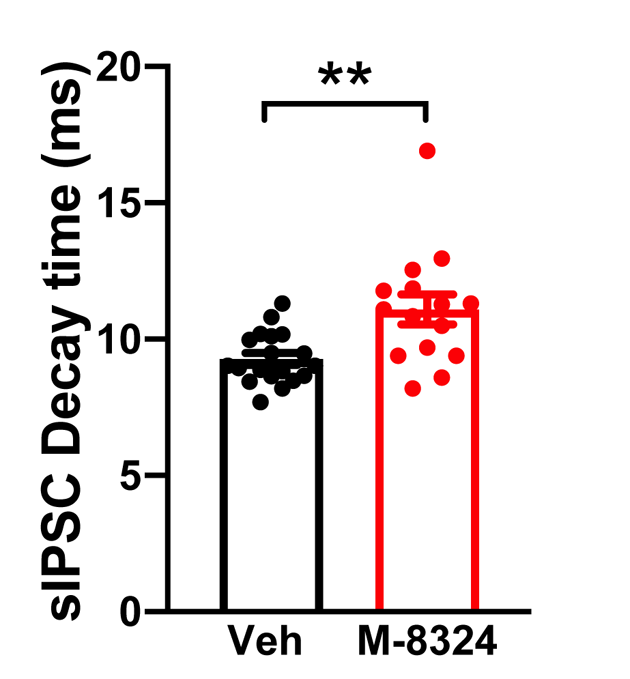

Supplement: Supplementary file 13 [file Image10.TIF]

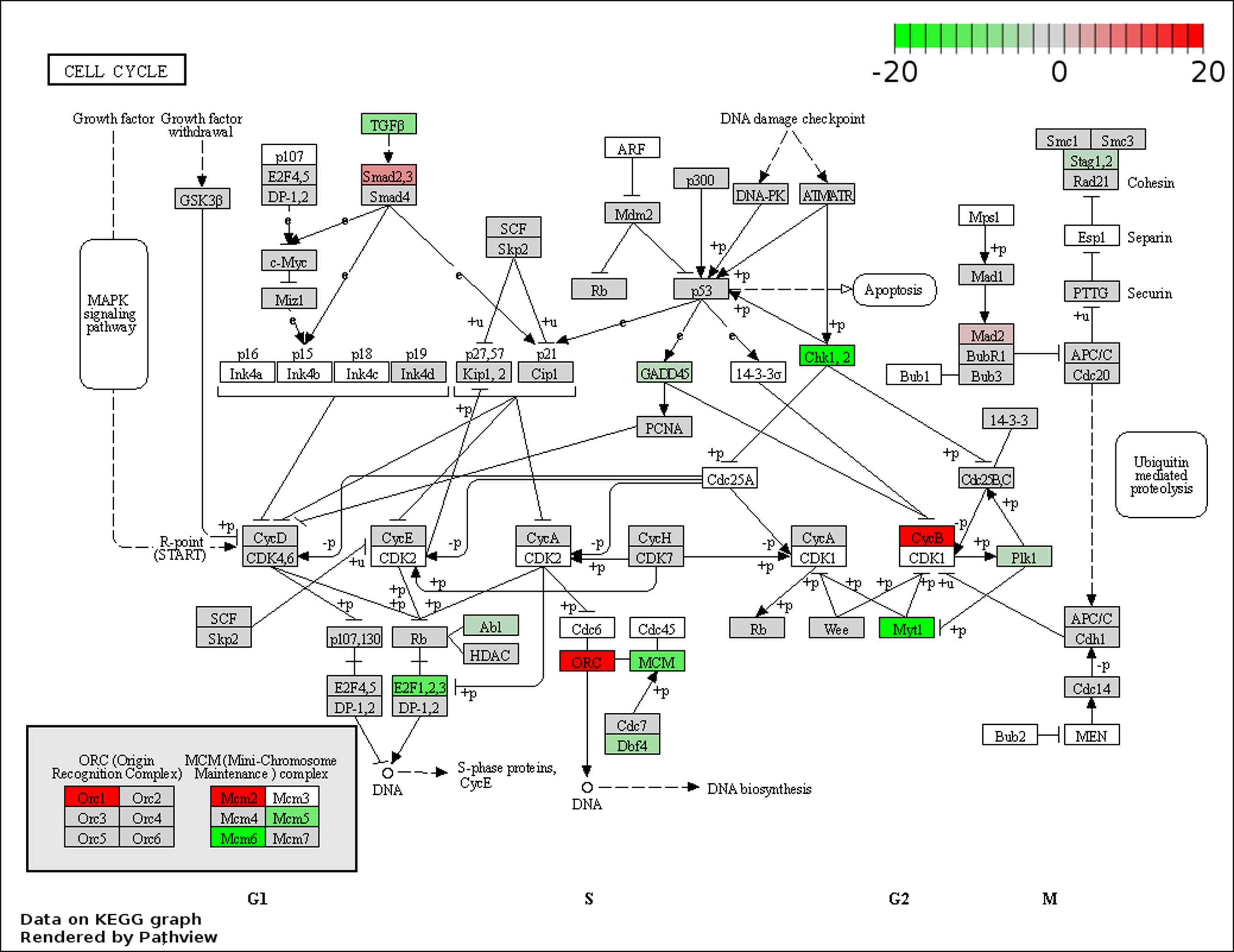

Supplement: Supplementary file 14 [file Image7.TIF]

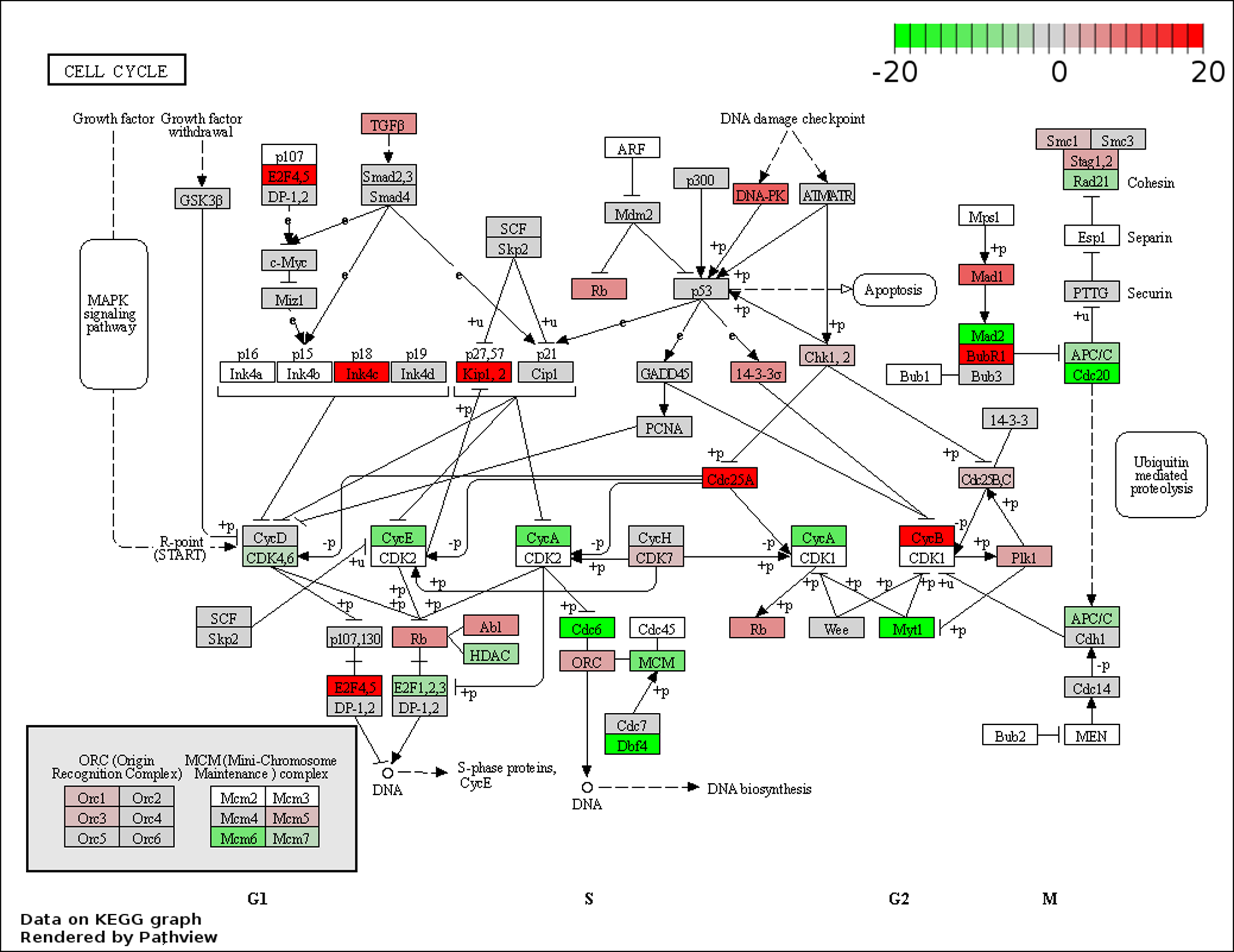

Supplement: Supplementary file 16 [file Image8.TIF]

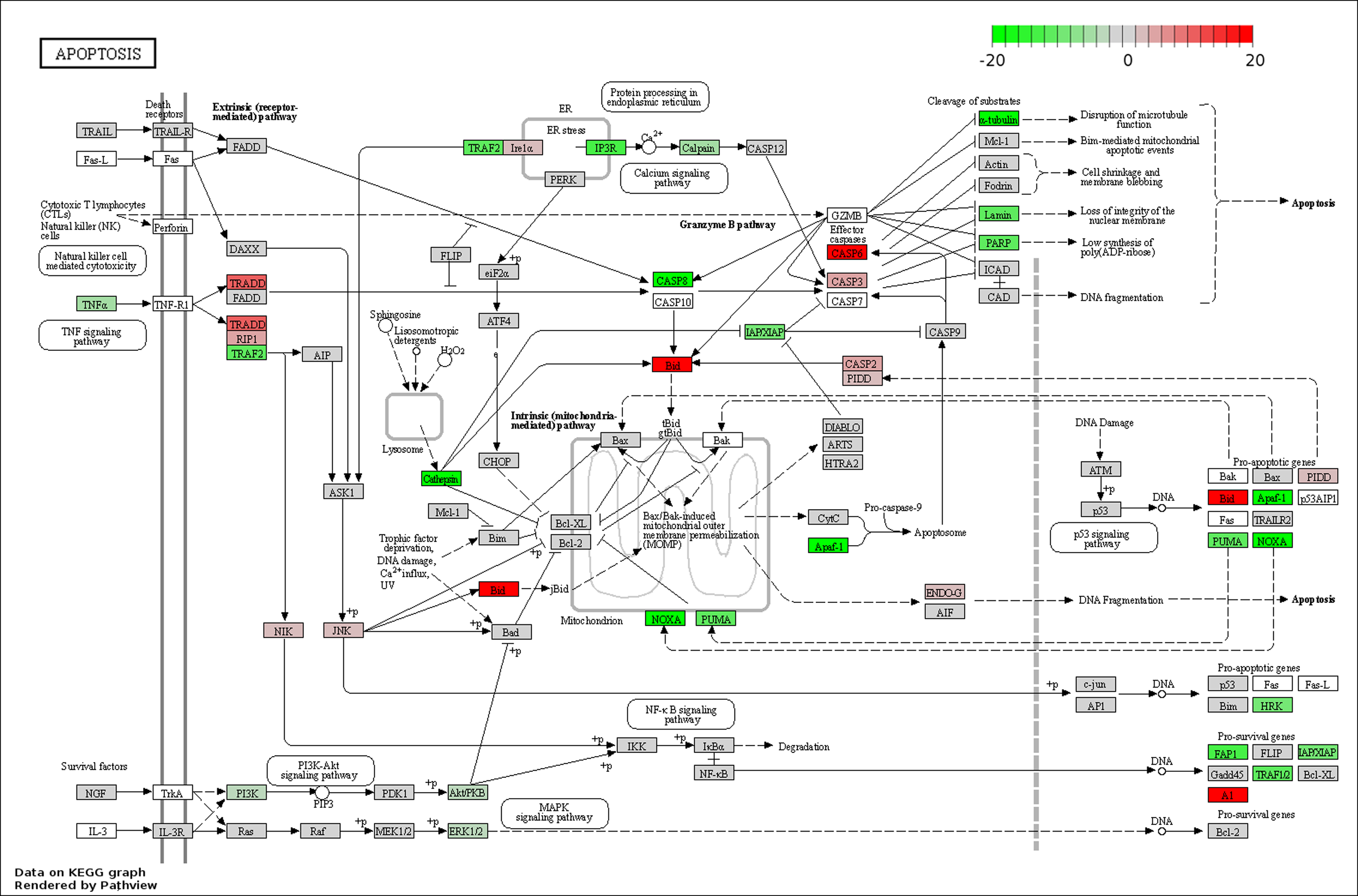

Supplement: Supplementary file 17 [file Image5.TIF]
