# Supplementary material for: Long-Term Enhancement of NMDA Receptor Function in Inhibitory Neurons Preferentially Modulates Potassium Channels and Cell Adhesion Molecules
Source: Front Pharmacol. 2022 Jan 4;12:796179. doi: 10.3389/fphar.2021.796179 (PMC8764260; doi:10.3389/fphar.2021.796179)
Supplement: Supplementary file 18 [file DataSheet1.docx]

**Supplemental Figures**

**Figure S1. Overview of DEGs after 7-day M-8324 treatment and between brain regions.**

1. Numbers of DEGs between treatments or between Au1 and PrL-PFC. **(B)** Venn analysis showing difference and similarity of DEGs after M-8324 treatment between Au1 and PrL-PFC. Red represents up-regulation and blue represents down-regulation. **(C)** Venn diagram showing genes unique or shared in Au1 and PrL-PFC.

**Figure S2. Analysis of GO enrichment of specifically and highly expressed genes in Au1 and PrL-PFC.**

Top 6 enriched in each term were graphed. The color represents the enriched *Q value*. The deeper the color, the smaller the Q-value. *, *Q value*<0.05.

**Figure S3. Expression heatmap of genes encoding GABA and Glutamate receptors and sodium and calcium channels between Au1 and PrL-PFC.**

**(A)** Comparisons of GABAR subunit expression levels between Au1 and PrL-PFC. **(B)** Comparisons of glutamate receptor subunit expression levels between Au1 and PrL-PFC. **(C)** Comparisons of Na^+^ channel subunit expression levels between Au1 and PrL-PFC. **(D)** Comparisons of Ca^2+^ channel subunit expression levels between Au1 and PrL-PFC. The redder the color the higher the expression level. Standardized method of the heatmaps: log (value + 1). *, *P*<0.05.

**Figure S4. Categories of region-specific or higher expressed CAMs in Au1 and PrL-PFC.**

The redder the color the higher the expression level. Standardized method in heatmap: log (value + 1).

**Figure S5. Impacts of M-8324 treatment on apoptosis pathways in Au1.**

The redder the color the greater the up-regulation, the greener the color the greater the down-regulation, and the white indicates the genes not been detected.

**Figure S6. Impacts of M-8324 treatment on apoptosis pathways in PrL-PFC.**

The redder the color the greater the up-regulation, the greener the color the greater the down-regulation, and white color indicates genes not detected.

**Figure S7. Impacts of M-8324 treatment on cell cycle pathways in PrL-PFC.**

The redder the color the greater the up-regulation, the greener the color the greater the down-regulation, and white color indicates genes not detected.

**Figure S8. Impacts of M-8324 treatment on cell cycle pathways in Au1.**

The redder the color the greater the up-regulation, the greener the color the greater the down-regulation, and white color indicates genes not detected.

**Figure S9. Gene expression profiles of calcium channels in PV-neurons after M-8324 treatment.**

**(A)** Heatmap of all detected calcium channel subunits in Veh and M-8324 groups in Au1and PrL-PFC. **(B)** A Venn diagram showing the similarities and differences in the Ca^2+^ channel subunits in Au1 and PrL-PFC after M-8324 treatment. Heatmap: the redder the color the higher the expression level. Standardized method in heatmap: log (value + 1). Red: up-regulation; Blue: down-regulation.

**Figure S10. A significantly larger decay time of sIPSCs in M-8324 group compared to Veh group.**

**Figure S11. Categories of “high-up” and “low-down” CAMs after M-8324 treatment in Au1 and PrL-PFC.**
